# Comparative Transcriptomic Analysis Reveals That Ethylene/H_2_O_2_-Mediated Hypersensitive Response and Programmed Cell Death Determine the Compatible Interaction of Sand Pear and *Alternaria alternata*

**DOI:** 10.3389/fpls.2017.00195

**Published:** 2017-02-15

**Authors:** Hong Wang, Jing Lin, Youhong Chang, Cai-Zhong Jiang

**Affiliations:** ^1^Institute of Horticulture, Jiangsu Academy of Agricultural Sciences/Jiangsu Key Laboratory for Horticultural Crop Genetic ImprovementNanjing, China; ^2^Department of Plant Sciences, University of California at DavisDavis, CA, USA; ^3^Crops Pathology and Genetics Research Unit, United States Department of Agriculture, Agricultural Research ServiceDavis, CA, USA

**Keywords:** sand pear, *Alternaria alternata* (Fr.) Keissler, hypersensitive response, programmed cell death, ethylene, hydrogen peroxide, antioxidant enzyme

## Abstract

A major restriction on sand pear (*Pyrus pyrifolia*) production is black spot disease caused by the necrotrophic fungus *Alternaria alternata*. However, the pear response mechanism to *A. alternata* is unknown at the molecular level. Here, host responses of a resistant cultivar Cuiguan (CG) and a susceptible cultivar Sucui1 (SC1) to *A. alternata* infection were investigated. We found that the primary necrotic lesion formed at 1 dpi and the expansion of lesions was aggressive in SC1. Data from transcriptomic profiles using RNA-Seq technology identified a large number of differentially expressed genes (DEGs) between CG and SC1 in the early phase of *A. alternata* infection. K-mean cluster and Mapman analysis revealed that genes involved in ethylene (ET) biosynthesis and ET signaling pathway, such as ACS, ACOs, and ERFs, and in hypersensitive response (HR) and programmed cell death (PCD) were significantly enriched and up-regulated in the susceptible cultivar SC1. Conversely, genes involved in response to hydrogen peroxide and superoxide were differentially up-regulated in the resistant cultivar CG after inoculation with the fungus. Furthermore, ET levels were highly accumulated in SC1, but not in CG. Higher activities of detoxifying enzymes such as catalases were detected in CG. Our results demonstrate that the ET-/H_2_O_2_-mediated PCD and detoxifying processes play a vital role in the interaction of pear and *A. alternata*.

## Introduction

In plant-pathogen interactions, plant response to pathogens is based on two main mechanisms including microbe-associated molecular patterns (MAMPs) and the adaptive immune system (Bonardi et al., 2011). The action of plant resistance (R) genes belongs to the adaptive immune system (Bonardi et al., 2011). After recognition of the pathogen, the plant initiates defense strategies against pathogen attack. These include the hypersensitive response (HR) and the programmed cell death (PCD) at the infection site (Greenberg and Yao, 2004), followed by the complicated defense response and metabolic changes in the surrounding tissues and distal un-infected parts (La Camera et al., 2004). One of the earliest events in the HR is a burst of reactive oxygen species (ROS) including superoxide (${\text{O}}_{2}^{-}$), hydroxyl radical (OH^−^) and subsequent accumulation of H_2_O_2_ (Lamb and Dixon, 1997). The infection of an avirulent *Pseudomonas syringae* strain stimulated the production of ${\text{O}}_{2}^{-}$ and H_2_O_2_, and induced defense-related gene expression and cell death in Arabidopsis (Alvarez et al., 1998). H_2_O_2_ accumulation associated with the HR was detected when lettuce cells were inoculated with *P. syringae* pv *phaseolicola* (Bestwick et al., 1997). Whether ROS plays positive or negative roles during the HR is dependent on the type of pathogen. For example, in the Arabidopsis-*P. syringae* system, the accumulation of H_2_O_2_ induces cell death and restricts lesion development (Alvarez et al., 1998). However, in necrotrophic pathogen systems, such as *Botrytis cinerea* and *Sclerotinia sclerotiorum*, pathogens proliferate on dead tissues caused by the generation of oxidative burst (Govrin and Levine, 2000).

Plant hormones such as salicylic acid (SA), jasmonic acid (JA), ethylene (ET), and ROS, play key roles in PCD, as well as in the activation of plant defense responses. SA is required in plant resistance associated with the hypersensitive cell death during plant–pathogen interactions (Greenberg, 1997; Alvarez, 2000). At infection sites, SA binds to NON-EXPRESSOR OF PATHOGENESIS-RELATED GENES 3 (NPR3; Fu et al., 2012) and mediates the degradation of the cell-death suppressor (NPR1), therefore facilitating the occurrence of PCD and local effector-triggered immunity (ETI; Gust and Nurnberger, 2012). NPR1 interacting with TGAs, bZIP transcription factors, directly promotes the expression of pathogenesis-related (PR) proteins such as PR1, BGL2, and PR5 (Glazebrook, 2001), and therefore limits growth of pathogens and contributes to resistance. Several studies suggest that SA-mediated signaling pathways are involved in resistance to biotrophic or hemibiotrophic pathogens (McDowell and Dangl, 2000). For example, Pto-mediated resistance against the hemibiotrophic pathogen *P. syringae* is SA-dependent in tomato (Ekengren et al., 2003).

Pathogens also elicit JA and ET pathways. Unlike the SA pathway, a JA/ET-dependent defense provides strong resistance against necrotrophic pathogens that benefit from host cell death (Grant and Lamb, 2006). JA and ET are considered to act synergistically in response to pathogens and activate defense-related gene expression in Arabidopsis (Thomma et al., 1999). ETHYLENE RESPONSE FACTOR 1 (ERF1) integrates ET and JA signaling pathways to regulate the expression of downstream defense-related genes (Lorenzo et al., 2003). Plant defense-related genes such as *Defensin* (*PDF1*) or *proteinase inhibitors I* and *II* (*PI I* and *PI II*) are known as indicators of the ET and JA responses (Penninckx et al., 1996). ET is also involved in the regulation of both the timing and degree of PCD during plant-pathogen interactions (Greenberg, 1997; Wang H. et al., 2013). The initiation of HR results in a large burst of ET (Boller, 1991). In addition, ET acts in concert with SA as a positive regulator of cell death progression in an Arabidopsis *vad1* (*vascular associated death 1*) mutant (Bouchez et al., 2007). Transgenic petunia plants over-expressing the *A. thaliana* ET receptor mutant *ethylene-insensitive1-1* (*etr1-1*) have inhibited expression of senescence-associated genes *PhCP8* and *PhCP10*, thereby retarding the senescence caused by *B. cinerea* infection (Wang H. et al., 2013). The role of an ET-dependent pathway has been elucidated in AAL-toxin induced cell death (Moore et al., 1999). However, whether ET is involved in plant-*Alternaria alternata* (Fr.) Keissler (AK-toxin) interactions is still largely unknown.

Pears (*Pyrus* spp.) are one of the most important fruit trees in Europe, East Asia, and North America (Terakami et al., 2007). Black spot disease, caused by the Japanese pear pathotype of *A. alternata* (Fr.) Keisser, is one of the most serious diseases in Asian pear cultivation (Terakami et al., 2007). *A. alternata* (Fr.) Keisser produces host-selective toxins, AK-toxin, resulting in necrosis and leaf fall, which seriously restrict fruits yield in Asian pears (Terakami et al., 2007). A sand pear cultivar, Sucui1 (SC1), is widely cultivated in the Yangtze River basin, mainly because its fruits have excellent flavor and a less rusty exocarp than its male parent Cuiguan (CG). However, SC1 displays much stronger susceptibility to *A. alternata* (Fr.) Keisser than its parent CG in the field. To better understand molecular mechanisms governing the susceptibility and compatible interaction of pear-*A. alternata*, the transcriptome dynamics of the diverse responses between the resistant (CG) and susceptible (SC1) pear cultivars to the pathogen *A. alternata* were investigated using the RNA-seq technology. Our results illustrated that the ET-/H_2_O_2_-mediated PCD and detoxification play a vital role in the interaction of pear and *A. alternata*.

## Materials and methods

### Plant materials

Seven-year-old “Cuiguan” (CG) and “Sucui 1” (SC1) sand pear trees [*Pyrus pyrifolia* (Burm.f.) Nakai; rootstock: *Pyrus betulaefolia* Burge] were grown at the Pear Germplasm Resource Preservation Center, Jiangsu Academy of Agricultural Sciences, Nanjing, China. Leaves were collected from the orchard for inoculation assays.

### Inoculation assays

*A. alternata* was grown on potato dextrose agar plates (Sigma-Aldrich, USA) at 25°C for 7 days (Suzuki et al., 2003). The conidial suspension was obtained through rinsing mycelia mats with distilled water. The spore concentration was 1.0 × 10^6^ sprores mL^−1^ for inoculation.

Adaxial epidermis of detached leaves was punctured by a 0.30 mm needle to form four infection sites along both sides of the center vein. Ten microliters of the inoculants were applied to each punctured site. Sterilized distilled water was used as a mock control (Wang H. et al., 2013). The inoculated and mock treated leaves were incubated at the same boxes at 25°C under dark conditions with 100% relative humidity. The fungal growth process was evaluated for disease severity and disease incidence every day after inoculation. At least 20 detached leaves were used for each treatment. The experiments were repeated five times. Leaves were pooled at 0, 1, 2, 3, and 5 days post inoculation (dpi) for RNA-seq analysis.

### RNA extraction, library construction, and sequencing

Ten leaves were pooled at a given time point and three independent biological replicates of every pool were used for RNA preparation, library construction and sequencing. Total RNA was extracted using the Trizol method (Invitrogen, USA), combined with Ambion RiboPure™ Kit (Ambion, USA) (Wang H. et al., 2013). Quality and quantity of RNA were determined using a NanoDrop 3100 spectrophotometer (Thermo Scientific, USA). mRNA was obtained using Sera-mag (Thermo Scientific, USA). The cDNA libraries were prepared according to the Illumina protocols. Fragments of about 300 bp were excised from agarose and enriched by PCR for 16 cycles. Finally, the cDNA libraries were sequenced using an Illumina HiSeq 2500 machine to perform 100 paired-end sequencing according to HiSeq 2500 User Guide.

### Sequence data processing and differential gene expression analysis

Clean data were obtained from raw data by removing adapter sequences, trimming reads with poly-N and low quality reads. Clean reads from all 30 samples (five time points, three biological replicates, and two cultivars) were pooled and the read counts were normalized for quantifying the gene expression level. Sequences were mapped to a pear reference genome (http://peargenome.njau.edu.cn) for further analysis.

Gene expression values of RNA-Seq data were obtained by FKPM (Trapnell et al., 2010). For each pear genotype, DEGs were generated by comparing multiple treatments or two treatments (pairwise analysis) with the cut off FDR ≤ 0.05 and Fold change [FC] ≥ 2.0 (Trapnell et al., 2010). K-means clustering was exploited to obtain knowledge about expression profiles throughout the five time points with two genotypes (Nham et al., 2015). Gene Ontology (GO; FDR < 0.01) was used to describe gene function. KEGG Ortholog database (KO; FDR < 0.01) was used to elucidate the pathways of DEGs (Shin et al., 2014). Diverse GO terms with similar expression patterns of CG and SC1 were compared and analyzed.

Mapman visualization was performed as described previously (Thimm et al., 2004) to identify specific and common genes involved in response of two genotypes to *A. alternata*. Contigs were uploaded into Mapman as described previously (Nham et al., 2015). Mapping files produced by Mecrator and gene expression changes were viewed in Mapman v.3.5.1R2 (Thimm et al., 2004).

### Hormone measurements

ET emission was monitored using a gas chromatograph with a flame ionization detector as previously described (Bashan, 1994). Infected leaves of CG and SC1 were placed into 50 ml volume closed glass vials and incubated at 28 ± 1°C for 24 h. ET production was measured. As a control, leaves inoculated with sterile distilled water were subjected to the same sampling procedures. The rate of ET was calculated according to previous report (Bashan, 1994). Each experiment was repeated three times with at least three biological replicates.

### H_2_O_2_, ${\text{O}}_{2}^{-}$ and antioxidant enzyme activity measurements

The level of H_2_O_2_ was measured according to Sagisaka (1976). Briefly, 0.5 g FW of detached leaves were homogenized in a pre-chilled mortar and pestle in liquid nitrogen with 5% cold trichloroacetic acid (TCA) and then centrifuged (Eppendorf, Hamburg, Germany) at 17,000 g for 10 min at 0°C. The supernatants (1.6 mL) were mixed with 0.4 mL of 5% TCA, 0.4 mL of 10 mM ferrous ammonium sulfate and 0.2 mL of 2.5 mM potassium thiocyanate and used to measure H_2_O_2_ levels. Activities of superoxide dismutase (SOD) and catalase (CAT) were assayed according to previous descriptions (Wang H. et al., 2013). Briefly, the ground tissues were incubated in the enzyme extraction buffer containing potassium phosphate (pH 7.5) for 0.5 h at 4°C. The extracted solutions were then centrifuged at 13,000 g for 20 min at 4°C. The supernatants were divided into two identical aliquots and supplemented with the reaction buffer either for SOD or CAT analysis. The level of ${\text{O}}_{2}^{-}$ was measured using the commercial kits (Catalog #: A052-1, Nanjing Jiancheng Bioengineering Institute, China) according to the manufacturer's instruction. Quantification of enzyme activities, levels of ${\text{O}}_{2}^{-}$ and H_2_O_2_ were carried out spectrophotometrically at 25°C with UV-VISO 2450 (Shimadzu, Kyoto, Japan). Five biological samples were used from each experiment.

### qRT-PCR analysis

Leaves were pooled at 0, 9, 24, and 48 h post inoculation (hpi) for quantitative real-time PCR (qRT-PCR) (ABI 7300; Applied Biosystem, Foster City, CA, USA) as previously described (Ma et al., 2015). Gene sequences were retrieved from the pear genome database. Specific primers were designed by the PRIMER 3 program and listed in Supplementary Table S1. The 2^−ΔΔCT^ method was used for evaluation of the relative levels of gene expression (Ma et al., 2015). A house-keeping gene, *GAPDH* (Glyceraldehyde 3-phosphate dehydrogenase), was used as an internal standard for normalization (Yang et al., 2015).

### Statistical analyses

All statistical analyses were performed using the SPSS package (Version 16.0; SPSS Inc., Chicago, IL, USA). One-way analysis of variance (ANOVA) was performed for experiments with one independent variable. Duncan's test was used if significant differences were found.

## Results

### Assessment of *A. alternata* growth in two different genotypes

**Figure 1:**
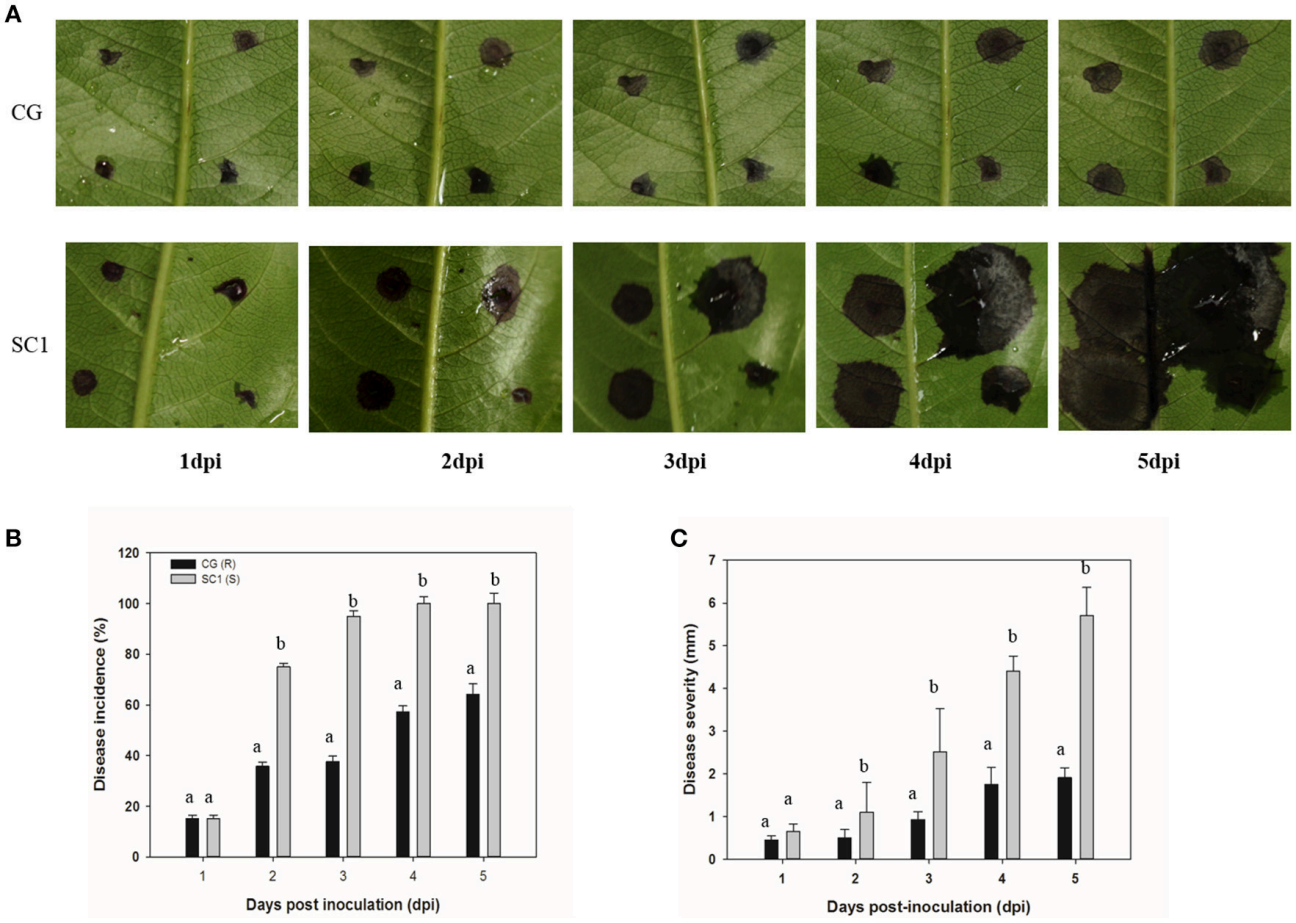
**Development of ***A. alternata*** on CG and SC1. (A)** Representative phenotypes of CG and SC1 inoculated with *A. alternata*. **(B)** Disease incidence (percentage of inoculation sites with expanding lesions) of the fourth leaves from the top on CG and SC1 branches *in vitro*. **(C)** Disease severity (diameter of expanding lesions) of the fourth leaves from the top on CG and SC1 branches *in vitro*. Mean values are shown from three independent biological replicates [error bars, ±standard error (SE)] containing at least 20 leaves (80 droplets) for every experiment. Different letters indicate significant differences between two genotypes at a given time point (*P* ≤ 0.05).

We found that two genotypes of SC1 and CG infected with *A. alternata* exhibited different progressions of leaf symptom development. The pear cultivar SC1 showed more severe disease symptoms of black spots (Figure 1A). The first symptom, necrotic spots, was observed at 1 dpi, followed by rapidly expanding lesions at 3 dpi and leaf soaking at 5 dpi. However, primary necrotic lesions in leaf tissue of its male parent CG formed at 1 dpi and spread very slowly at 5 dpi. The disease incidence in leaf tissue of CG was < 70%, whereas it was ~100% in SC1 leaves (Figure 1B). The disease severity was significantly greater in SC1 leaves (3.47 cm at 5 dpi) than CG leaves (1.67 cm at 5 dpi; Figure 1C).

### Sequence identity and expression analysis of RNA-seq transcriptome data

The raw reads were trimmed by removing adaptor sequences, empty reads and low-quality sequences to produce the clean reads. As a result, more than 97% clean ratio for each sample was obtained (Table 1). The majority of clean reads (more than 65%) were successfully mapped to the pear reference genome (Wu et al., 2013). As evidence of disease development in pear cultivars of SC1 and CG, the clean reads were also mapped to *A. alternata* genome (Dang et al., 2015). An insignificant proportion of reads were mapped to *A. alternata* reference genomes, for example, 0.2263% for SC1 and 0.2596% for CG in the symptomatic samples (5 dpi), compared with 0.0063% for SC1 and 0.0053% for CG in non-symptomatic leaves (0 dpi; Table 1).

**Table 1:** **Mapping characteristics of CG, SC1, and ***A. alternata*** reads to the reference genomes in the thirty samples at different time points after inoculation**.

**Sample**	**Raw reads**	**Clean reads**	**Reads mapped Pear genome**	**Reads mapped *Alternaria* genome**
C0	42,426,142	41,508,319	27,268,734 (68.22%)	2,019 (0.0053%)
C1	47,619,512	46,601,111	30,788,480 (68.21%)	14,328 (0.0320%)
C2	45,182,075	44,201,272	28,825,665 (68.00%)	9,849 (0.0240%)
C3	42,782,298	40,718,867	27,383,506 (68.14%)	35879 (0.1210%)
C5	41,217,044	39,267,755	27,138,755 (67.35%)	82489 (0.2596%)
S0	48,690,054	47,668,210	30,059,693 (65.75%)	2605 (0.0063%)
S1	46,843,642	45,854,345	27,930,754 (65.74%)	8409 (0.0196%)
S2	45,106,658	44,132,629	28,423,723 (66.22%)	20278 (0.0766%)
S3	39,591,996	38,006,025	28,025,023 (66.23%)	85039 (0.2001%)
S5	40,196,180	38,494,785	28,629,681 (65.45%)	69859 (0.2263%)

**Figure 2:**
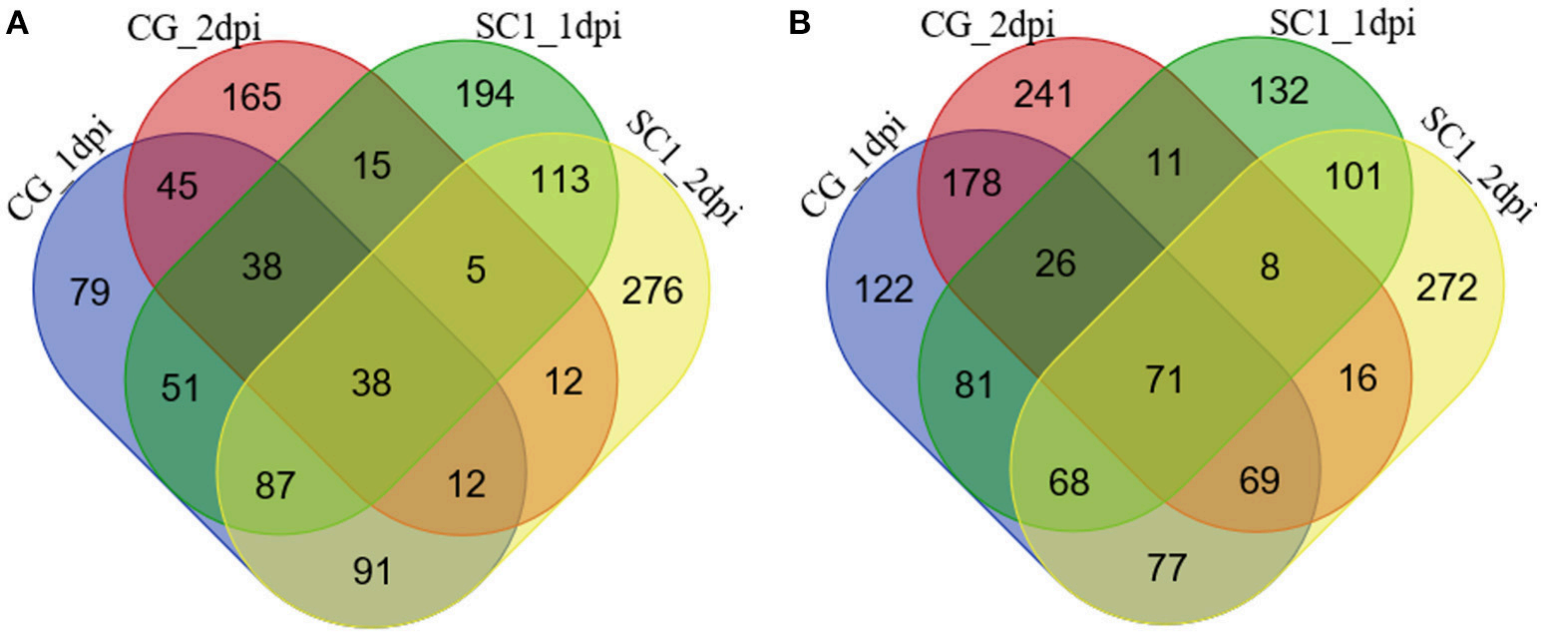
**Unique and shared differentially up-regulated (A)** and down-regulated **(B)** unigenes in CG and SC1 at given time points. Blue, CG 1 dpi; purple, CG 2 dpi; green, SC1 1 dpi; yellow, SC1 2 dpi.

The significantly increased numbers of DEGs between the two genotypes before and after 3 dpi were determined (Supplementary Table S2), ranging from 1188 (C0–S0), 1154 (C1–S1), and 1414 (C2–S2) to 3114 (C3–S3) and 2623 (C5–S5). The number of DEGs between the early stage (0–2 dpi) and later stage (3–5 dpi) in the same genotype, was significantly increased (Supplementary Table S2), ranging from 951 (C1–C0) and 1134 (C2–C0) to 5834 (C3–C0) and 6000 (C5–C0) in the CG leaves, and from 1040 (S1–S0) and 1319 (S2–S0) to 7222 (S3–S0) and 7526 (S5–S0) in the SC1 leaves, respectively. Further analysis identified unique and shared DEGs between these two genotypes (Figure 2). The general biological functions of these DEGs were analyzed using Mapman. The results showed that the highest numbers of DEGs were assigned to biological processes of protein (11.65%), RNA (9.61%), signaling (5.96%), and misc (5.11%). Other over-represented categories of biological processes included biotic stress (4.62%), transport (3.98%), cell (2.86%), hormone metabolism (2.64%), development (2.59%), and secondary metabolism (2.34%; Figure S1).

### Time-course expression profiles analysis

**Figure 3:**
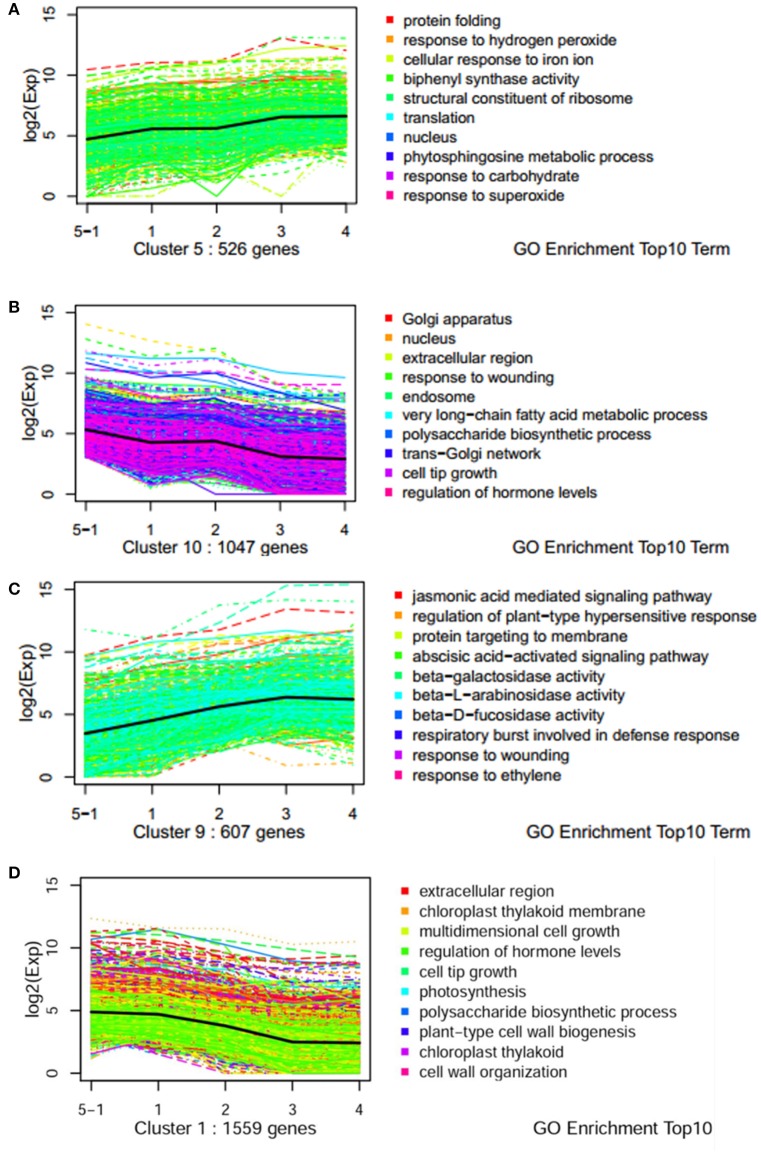
**Expression profiles of genes with top 10 GO term across five stages of two genotypes by k-mean analysis. (A)** Up pattern in CG; **(B)** Down pattern in CG; **(C)** Up pattern in SC1; **(D)** Down pattern in SC1.

To understand the molecular mechanisms governing the susceptibility of two genotypes, dynamic expression trends of DEGs were analyzed using K-mean clustering. Ten profiles were obtained for dissecting the expression patterns (Figures S1, S2). Dynamic processes of plant response to pathogen were illuminated by significantly enriched GO terms. For example, the terms of “phytosphingosine metabolic process,” “response to hydrogen peroxide,” and “response to superoxide/carbohydrate” were enriched in an up-regulated pattern in the resistant genotype CG leaves (Figure 3A). Biological processes such as “polysaccharide biosynthetic processes,” “response of hormone levels,” and “very long-chain fatty acid metabolic” were enriched in a down-regulated pattern in CG (Figure 3B). However, the terms of “jasmonic acid mediated signaling pathway,” “response to ethylene/wound,” “ABA-activated signaling pathway,” “regulation of plant-type hypersensitive response,” and “respiratory burst involved in defense response” were enriched in an up-regulated pattern in the susceptible genotype SC1 leaves (Figure 3C). Biological processes including “photosynthesis,” “polysaccharide biogenesis,” “plant-type cell wall biogenesis,” and “cell wall organization” were enriched in a down-regulated pattern in SC1 (Figure 3D). In addition, down-regulated expression patterns of cellular components were observed, such as “Golgi apparatus,” “nucleus,” “endosome” and “trans-Golgi network” in infected leaves of CG and “chloroplast thylakoid membrane,” and “chloroplast thylakoid” in infected leaves of SC1 (Figures 3B,D).

### Genes involved in regulation of hypersensitive response and programmed cell death

K-mean analysis suggested that genes related to HR, PCD, hormone biosynthesis, and signaling pathways, and transcription factors were significantly enriched after inoculation (Figure 3). In addition, genes related to defense responses displayed either induced or repressed expression patterns in two different genotypes (Figure 3 and Figure S1). Therefore, genes classified in “regulation of plant-type hypersensitive response” and “programmed cell death” were further analyzed. Of 32 DEGs related to these two biological processes, a gene encoding patatin-like protein 2 (PLP2) (Pbr004131.1) was continuously and differentially induced in the susceptible genotype SC1 but not in the resistant genotype CG (Table 2). Kunitz trypsin inhibitor (KTI) (Pbr037895.1), WRKY 40 (Pbr022408.1), PGIP (Pbr030600.1 and Pbr030601.1), HSL1 (Pbr006072.1), UGT85A (Pbr007515.1 and Pbr007514.1), receptor-like protein kinase HAIKU2 (Pbr040910.1), cationic amino acid transporter 1-like (CAT1) (Pbr037476.1), NAC25 (Pbr004500.1) were significantly and continuously induced in infected leaves of SC1 but not CG (Table 2). SAG20 (senescence associated gene, Pbr010964.1) was induced in SC1 but not CG. Similarly, a gene encoding senescence-associated protein SAG12 (pbr006728.1) was significantly up-regulated at 3 dpi of SC1 but not CG (Table 2).

**Table 2:** **Unigenes associated with related biological function exhibiting a Log2 FC ≥ 1 and ***p*** ≤ 0.05 in at least one transition**.

**Gene ID**	**CG (Resistant)**			**SC1 (Susceptible)**			**Description**
	**FCC1/C0**	**FCC2/C0**	**FCC3/C0**	**FCS1/S0**	**FCS2/S0**	**FCS3/S0**	
**HYPERSENSITIVE RESPONSE AND PROGRAMMED CELL DEATH**							
pbr010964.1	0.84	0.20	−1.36^*^	2.35^*^	0.70	−0.06	SAG20
pbr006728.1	–	–	–	−0.04	0	2.60^*^	SAG12
Pbr012334.1	1.37	−0.68	−3.37	6.98	9.76^*^	10.96^*^	PLP2
Pbr004131.1	2.59^*^	1.78^*^	−1.92^*^	5.60^*^	8.25^*^	8.97^*^	PLP2
Pbr012337.1	2.10^*^	0.33	−2.09	7.59	10.45^*^	12.67^*^	PLP2
Pbr035719.1	1.44	0.37	0.44	3.15	3.15	5.27^*^	SYP21
Pbr037895.1	1.74^*^	0.50	−1.14^*^	1.47^*^	3.59^*^	6.56^*^	KTI
Pbr030600.1	−1.10^*^	−4.66^*^	−4.87^*^	3.13^*^	3.98^*^	3.96^*^	PGIP
Pbr030601.1	−1.24^*^	−5.03^*^	−6.96^*^	4.08^*^	4.74^*^	5.88^*^	PGIP
Pbr010566.1	0.51	−1.18	−1.62	2.81	3.17	6.50^*^	PDR1
Pbr006072.1	0.64	−1.12^*^	0.09	1.12^*^	1.30^*^	1.54^*^	HSL1
Pbr008587.1	1.51^*^	0.21	−1.68^*^	0.35	1.32	2.63^*^	HSL1
Pbr015532.1	−0.30	−1.55	−0.06	0.75	1.57	3.13^*^	HSL1
Pbr008766.1	1.24	−1.13	0.16	1.29	2.22^*^	2.65^*^	HSL1
Pbr007513.1	0.71	−0.19	−1.46^*^	1.56	2.67	2.49^*^	UGT85A24A5-like
Pbr007515.1	0.96	−0.59	1.59	2.57^*^	4.18^*^	7.09^*^	UGT85A24A5-like
Pbr007514.1	0.77	−0.54	−0.32	1.66^*^	2.98^*^	5.56^*^	UGT85A23
Pbr021700.1	0.72	0.32	−1.34^*^	0.26	1.56^*^	2.33^*^	UGT85A24
Pbr040910.1	0.26	−1.26^*^	−0.51	1.81^*^	1.20^*^	1.98^*^	HAIKU2
Pbr037476.1	1.08^*^	−0.27	0.11	2.17^*^	2.09^*^	2.63^*^	CAT1
Pbr003247.1	0.06	−0.27	−1.24	1.04^*^	0.79	1.30^*^	ARG2-like
Pbr019124.1	2.11	0.86	4.04^*^	1.56	2.90	5.60^*^	ABCG36
Pbr026949.2	0.10	−0.63	0.50	0.60	2.28^*^	4.49^*^	NAC25-like
Pbr004500.1	0.85	0.64	0.26	1.20^*^	1.03^*^	1.46^*^	NAC25-like
Pbr004703.1	2.97	1.11	−0.20	1.71	3.11	4.95^*^	RIP3
**RESPONSE TO HYDROGEN PEROXIDE AND SUPEROXIDE**							
Pbr027587.1	1.13^*^	1.69^*^	2.29^*^	0.91	0.15	2.17^*^	MBF1C
Pbr016628.1	1.68^*^	2.78^*^	2.88^*^	1.36^*^	1.08	1.00	HSP20
Pbr032362.1	0.26	0.96	0.32	0.95	0.51	2.23^*^	HSP70
Pbr040066.1	1.79	1.63	1.98	2.14^*^	1.16	2.65^*^	HSP22
Pbr003834.1	0.53	0.57	1.11^*^	0.60	0.39	1.45^*^	RAB11C
Pbr028049.1	0.26	0.44	1.09	0.43	0.76	2.38^*^	CINV2
Pbr028718.1	1.21	1.48	2.75	3.04^*^	−0.16	2.66^*^	dnaJ6-like
Pbr031275.1	0.62	0.83	1.36^*^	0.40	−0.14	0.84	SRO3
Pbr013783.1	0.68	1.24^*^	0.83	0.63	0.36	0.91	RCD1
Pbr025956.1	0.52	0.61	1.27^*^	0.93	0.24	1.17^*^	RCD1
Pbr016212.1	0.31	0.77	1.20	0.72	0.40	1.91^*^	dnaJB13
Pbr027173.1	0.48	0.36	1.37^*^	1.74^*^	0.95	2.65^*^	GIGANTEA-like
Pbr035808.1	0.45	0.48	1.16^*^	0.04	0.26	1.33^*^	AHSA1
Pbr036899.1	0.49	0.33	1.55^*^	0.49	0.09	2.01^*^	EXO70B1
Pbr033477.1	2.00	0.34	2.40^*^	1.61	1.96	5.15^*^	ABCB5
Pbr019106.1	1.12	2.07	3.75^*^	−0.51	−1.09	2.55	PTP1
Pbr014824.1	2.88^*^	3.07^*^	2.79^*^	0.26	1.07^*^	−1.10	GRXS10
Pbr016271.1	0.41	1.01^*^	0.50	0.42	0.38	−0.63	GRXS17
Pbr016203.1	−0.26	−0.68	−1.12^*^	1.28	1.78	3.21^*^	GRXS9

*HAIKU2, receptor-like protein kinase; CAT1, cationic amino acid transporter 1-like; RIP3, ras-interacting protein; indole-3-acetic acid-induced protein ARG2-like; PTP1-like, protein-tyrosine-phosphatase*.

* *The gene was differentially expressed in the correspondent pairwise analysis (p ≤ 0.05)*.

### Genes involved in response to hydrogen peroxide, superoxide, and carbohydrate

K-mean analysis suggested that genes related to the responses to hydrogen peroxide, superoxide and carbohydrate were significantly up-regulated in the resistant genotype CG but not in the susceptible genotype SC1 (Figure 3A). The expression patterns of genes enriched in these GO terms were further analyzed (Table 2). The expression of majority of the genes (20/21) enriched in these GO terms was induced in the resistant genotype CG (Table 2). Of those genes, MULTIPROTEIN BRIDGING FACTOR 1c (MBF1c) (Pbr027587.1), Heat shock proteins (HSP20) (Pbr016628.1), and Glutaredoxins10 (GRXS10) (Pbr014824.1) were significantly and continuously up-regulated in the resistant genotype CG but not in the susceptible genotype SC1 (Table 2).

### Genes involved in hormone biosynthesis and signaling

GO terms such as “JA, ABA-mediated signaling pathway” and “response wounding and ET” were clustered in significantly up-regulated expression patterns in SC1 (Figure 3C) and down-regulated expression patterns in CG (Figure 3B). We further dissected the expression patterns of the genes involved in the biosynthesis and signaling pathways for plant hormones ET, SA, JA, and ABA during plant–pathogen interactions.

**Figure 4:**
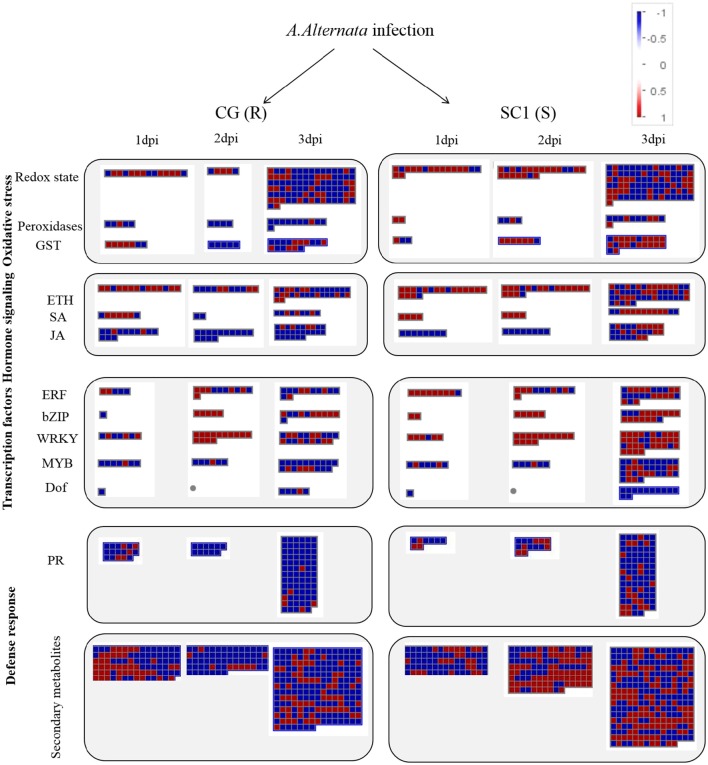
**Display of transcriptional response of CG and SC1 to biotic stress**. Significantly differentially expressed genes (Log_2_ fold changes [FC] ≥ 1, FDR ≤ 0.05) were visualized using Mapman software and organized into functional categories (BINs). Blue indicates a decrease and red an increase gene expression (see color set scale on top right corner). Detailed information on each gene and its expression level were listed in Tables 2–5 and Supplementary Table S3.

A predominance of up-regulated genes related to the ET biosynthesis and signaling pathways was found in the susceptible genotype SC1 (Figure 4 and Table 3). Transcript abundances were highly accumulated in the infected leaves of SC1 for most ET biosynthesis-related genes i.e., ACS (Pbr032688.1), ACO1-like (Pbr023057.1, Pbr023059.1, Pbr0326881, Pbr031954.1, Pbro15589.1), ACO4-like (Pbr012109.1, Pbr015355.1, and Pbr021636.1), ACO5-like (Pbr040048.1, Pbr040049.1, and Pbr013513.1) as well as for ET signaling components such as ETR2 (Pbr002199.1) and ERS1 (Pbr022706.1). Notably, ACS (Pbr032688.1), ACO1-like (Pbr023057.1 and Pbr023059.1), and ACO5-like (Pbr013513.1) were sustained and differentially induced in the leaves of the susceptible genotype SC1 but not in the resistant genotype CG (Table 3).

**Table 3:** **Unigenes associated with hormone biosynthesis and signaling pathway exhibiting a Log2 FC ≥ 1 and ***p*** ≤ 0.05 in at least one transition**.

**Gene ID**	**CG (Resistant)**			**SC1 (Susceptible)**			**Description**
	**FCC1/C0**	**FCC2/C0**	**FCC3/C0**	**FCS1/S0**	**FCS2/S0**	**FCS3/S0**	
**HORMONE BIOSYNTHESIS AND SIGNALING PATHWAY–ETHYLENE PATHWAY**							
Pbr032688.1	1.99	1.65	0.41	2.91	6.58^*^	656.2^*^	ACS
Pbr023057.1	1.18^*^	0.38	−0.11	1.06^*^	1.50^*^	2.42^*^	ACO1-like
Pbr023059.1	1.26^*^	0.45	0.77	1.10^*^	1.71^*^	3.74^*^	ACO1-like
Pbr032688.1	2.00	1.65	−1.27	2.91	6.58^*^	9.36^*^	ACO1-like
Pbr031954.1	1.49^*^	−3.94^*^	−5.57	1.96	4.26^*^	1.42	ACO1-like
Pbr015589.1	0.55	−1.27	−4.40^*^	5.69^*^	3.97	1.14	ACO1-like
Pbr005179.1	−1.22^*^	−1.89^*^	−1.85^*^	−1.08^*^	−1.55^*^	−1.83^*^	ACO3-like
Pbr012109.1	0.33	−0.59	−0.52	−0.13	0.81	0.79	ACO4-like
Pbr015355.1	1.73	−0.46	0.97	2.81^*^	4.38^*^	4.67^*^	ACO4-like
Pbr021636.1	1.80^*^	−2.46^*^	−4.29^*^	1.08	3.78^*^	2.65^*^	ACO4-like
Pbr040048.1	1.17	2.09^*^	0.77	0.42	2.43^*^	2.43^*^	ACO5-like
Pbr040049.1	1.95^*^	2.61^*^	1.25	0.53	2.76^*^	2.47^*^	ACO5-like
Pbr013513.1	0.97	0.93	−0.36	1.80^*^	2.35^*^	1.81^*^	ACO5-like
Pbr023072.1	1.92^*^	0.83	−1.10	1.37^*^	0.42	−3.12^*^	ETR2
Pbr022706.1	1.99^*^	0.01	−1.39^*^	1.24^*^	1.23^*^	0.51	ERS1
Pbr026603.1	0.65	0.82	1.22^*^	0.72	0.33	0.88	EIN3-like
Pbr033210.1	0.89	0.93	1.47^*^	0.61	0.35	0.63	EIN3-like
**HORMONE BIOSYNTHESIS AND SIGNALING PATHWAY–SA PATHWAY**							
Pbr027953.1	−0.49	−1.20^*^	−1.36^*^	0.19	1.13^*^	1.87^*^	PAD4
Pbr025644.1	1.78^*^	−0.29	2.40^*^	1.62^*^	2.60^*^	5.04^*^	UGT72E2
Pbr025645.1	1.89	−0.36	1.54	1.32	2.91^*^	6.15^*^	UGT72E2
Pbr025643.1	–	−0.38^*^	1.68	0.17	0.43	2.09^*^	UGT74E1
Pbr002691.1	–	0.07	−0.04	0.40	1.95^*^	2.08^*^	UGT73B4
Pbr007397.1	1.06^*^	−1.35	−2.10	1.48	4.06^*^	6.25^*^	UGT74E2
Pbr020349.1	0.84	−0.91	−2.39^*^	1.93^*^	2.55^*^	5.04^*^	SAM
Pbr025059.1	1.67	−1.04	3.32	2.23^*^	0.30	10.02^*^	SABP2
Pbr031493.1	−0.07	0.33	0.83	1.69	−0.41	1.70	NPR3-like
**HORMONE BIOSYNTHESIS AND SIGNALING PATHWAY—JA PATHWAY**							
Pbr020412.1	3.07^*^	0.07	−1.93	3.85	8.25^*^	9.25^*^	LOX1-like
Pbr020414.1	0.01	−1.76^*^	−1.40^*^	0.83	1.07	0.64	LOX1-like
Pbr020415.1	0.71	0.32	−0.96	1.37	3.16^*^	4.30^*^	LOX1-like
Pbr004969.1	−2.33^*^	−1.11^*^	−4.67^*^	−1.28^*^	−1.44^*^	−7.30^*^	LOX2
Pbr005350.1	−1.84^*^	−1.83^*^	−0.95	−1.00	−1.25^*^	−1.16^*^	LOX3
Pbr006204.1	−2.48^*^	−2.24^*^	−4.20^*^	−1.60	−0.96	−3.46^*^	AOS-like
Pbr006205.1	−2.37^*^	−2.00^*^	−1.90^*^	−1.69	−1.22	−0.41	AOS-like
Pbr004466.1	4.73	0.09	−0.26	−1.57	2.72^*^	0.18	AOS-like
Pbr008503.1	3.85	−0.37	1.18	−2.14	2.38^*^	4.76^*^	AOS-like
Pbr013257.1	−1.80^*^	−2.21^*^	−3.25^*^	−0.66	0.21	0.29	AOC4-like
Pbr027476.1	−0.98	−1.17^*^	−2.22^*^	−1.14^*^	−0.92	−3.19^*^	AOC4-like
Pbr030638.1	−6.82^*^	−5.18^*^	−5.78^*^	−3.10^*^	−1.95^*^	0.59	AOC4-like
Pbr005926.1	−5.84^*^	−2.95^*^	–	−2.08	−3.40	−3.79	JMT
Pbr027730.1	−2.30	−2.09^*^	−2.02^*^	−1.57^*^	−0.99	−0.12	TIFY 10A
Pbr039229.1	−0.41	−1.52^*^	−4.04^*^	1.81	3.38^*^	4.59^*^	TIFY10B-like
Pbr012103.1	−2.19^*^	−1.55	−3.56^*^	−1.61	−1.39	−3.63^*^	TIFY6A-like
Pbr037418.1	−4.51^*^	−3.71^*^	−6.56^*^	−3.08^*^	−2.79^*^	−4.42^*^	TIFY10A-like
**HORMONE BIOSYNTHESIS AND SIGNALING PATHWAY—ABA PATHWAY**							
Pbr010367.1	2.21^*^	0.95	−2.02	1.30	1.61^*^	1.79^*^	HVA22like
Pbr005978.1	1.12^*^	−0.18	−0.39	0.94	1.73^*^	2.51^*^	AAO1-like
Pbr008947.1	1.08^*^	−0.09	0.32	0.41	0.90	−0.31	AAO1-like
Pbr029414.1	1.06	−0.66	−3.60	2.43^*^	0.11	−4.40	ABA 8′-hydroxylase
Pbr019415.1	0.48	−0.04	0.16	2.96^*^	2.44^*^	3.62^*^	PYL4-like
Pbr028222.1	0.47	0.33	0.99	4.22^*^	3.47^*^	4.46^*^	PYL4-like
Pbr027457.1	0.93	0.34	0.42	0.98	0.92	1.25^*^	PYL9

* *The gene was differentially expressed in the correspondent pairwise analysis (p ≤ 0.05)*.

A predominance of down-regulated genes was found in the JA pathway in CG and SC1 (Figure 4 and Table 3). For example, genes encoding LOX2 (Pbr004541.1, Pbr004568.1 and Pbr023784.2), LOX3 (Pbr005350.1), AOS (Pbr006204.1 and Pbr006205.1), AOC (Pbr013257.1, Pbr027476.1 and Pbr030638.1), an OPR3 (Pbr041531.1), S-adenosyl-L-methionine (JMT, Pbr005926.1), three JAZ (Pbr027730.1, Pbr037418.1 and Pbr012103.1) were down-regulated in both CG and SC1 (Table 3).

*PAD4* (Phytoalexin Deficient 4, Pbr027953.1) was differentially up-regulated after 2 dpi in the susceptible genotype SC1 but down-regulated in the resistant genotype CG (Table 3). The differential expression of *ICS1* (isochorismate synthase 1, Pbr011477.1) and *EDS1* (Enhanced Disease Susceptibility 1) was not observed in either SC1 or CG (Supplementary Table S3).

Based on K-means analysis, the ABA-activated signaling pathway was up-regulated in SC1 but down-regulated in CG (Figure 3 and Figures S1, S2). Genes enriched in this GO term were further analyzed. Of seven DEGs, only genes encoding PYL4-like (Pbr019415.1 and Pbr028222.1) were differentially induced in the susceptible genotype SC1 (Table 3). These results suggested that ABA, JA, and SA pathways were triggered in the early stage of inoculation neither in SC1 nor CG.

### Transcription factors analysis

In the category of “response to biotic stresses,” transcripts of the AP2/EREBP and WRKY family members were the most abundant (Figure 4). To identify key genes that regulate pear response to *A. alternata*, we carried out a detailed analysis on these two TFs using Heat Map (Figures S3, S4) and Mapman (Figure 4 and Table 4). Of those, 21 DEGs were identified as ERFs in the transcriptome data (Figure S3). Genes encoding ERF2-like (Pbr035775.1), ERF1-like (Pbr001363.1), ERF113-like (Pbr029841.1), PTI (Pbr016185.1), and RAP2.3 (Pbr012024.1) were differentially up-regulated in the susceptible genotype SC1 leaves (Table 4). Approximately 25 WRKY genes were differentially expressed (Figure S4). A gene encoding WRKY40 (Pbr022408.1) was differentially up-regulated in the susceptible genotype SC1 leaves (Table 4).

**Table 4:** **Unigenes associated with TFs exhibiting a Log2 FC ≥ 1 and ***p*** ≤ 0.05 in at least one transition**.

**Gene ID**	**CG (Resistant)**			**SC1 (Susceptible)**			**Description**
	**FCC1/C0**	**FCC2/C0**	**FCC3/C0**	**FCS1/S0**	**FCS2/S0**	**FCS3/S0**	
**TRANSCRIPTION FACTORS-AP2/EREBP**							
Pbr017391.1	0.58	0.20	0.78	−0.53	−1.49^*^	−1.51^*^	ERF4-like
Pbr000396.1	−1.15	−1.79^*^	−4.60^*^	−2.04^*^	−2.08^*^	−4.39^*^	ERF12-like
Pbr001362.1	1.09^*^	0.53	−0.56	0.81	−0.41	−1.54^*^	ERF105-like
Pbr037414.1	−0.45	0.68	0.04	−1.64^*^	−4.28^*^	−1.59^*^	ERF017-like
Pbr019669.1	0.49	1.71^*^	2.29^*^	1.59	0.98	2.82^*^	ERF011
Pbr013255.1	0.67	0.03	0.68	1.73^*^	0.82	1.99^*^	RAV1-like
Pbr027478.1	−0.12	−1.05^*^	−0.99	1.10^*^	−0.23	−0.18	RAV1-like
Pbr016185.1	0.44	−0.88	−0.07	1.00^*^	1.09^*^	3.37^*^	PTI
Pbr004315.1	−2.94^*^	−2.28^*^	–	2.21	2.93	4.05	ERF1B-like
Pbr035775.1	0.43	−0.71	−0.93	2.17^*^	1.97^*^	1.97^*^	ERF2-like
Pbr037846.1	0.14	−1.15	−0.33	1.46	3.65^*^	4.05^*^	ERF113-like
Pbr030542.1	0.04	−1.79^*^	−0.68	1.38^*^	1.83^*^	1.17	ERF1B-like
Pbr001363.1	1.22	−0.56	0.52	2.95^*^	1.86^*^	2.31^*^	ERF1-like
Pbr030542.1	0.04	−1.79^*^	−0.68	1.38^*^	1.83^*^	1.17	ERF1B-like
Pbr023899.1	−0.10	−0.60	−0.71	1.10	0.72	1.60	ERF2-like
Pbr029841.1	−0.37	−1.87^*^	−0.70	2.41^*^	3.15^*^	3.67^*^	ERF113-like
Pbr012024.1	2.15^*^	1.14	−0.05	1.35^*^	2.24^*^	2.69^*^	RAP2-3
Pbr001361.1	1.16^*^	0.32	−2.47^*^	2.46^*^	1.60^*^	−0.58	ERF107-like
Pbr013149.1	1.05^*^	0.07	−0.66	1.05^*^	1.32^*^	0.44	PTI
Pbr007473.1	0.86	0.38	−0.70	1.33^*^	1.46^*^	0.34	ERF060
**TRANSCRIPTION FACTORS–WRKY**							
Pbr018160.1	0.64	0.27	1.57^*^	1.11^*^	0.46	1.49^*^	WRKY11
Pbr037640.1	−1.28^*^	−1.48^*^	−1.04^*^	−0.71	−0.35	−0.64	WRKY11
Pbr018132.1	0.68	0.27	−1.26^*^	1.14^*^	0.49	−0.09	WRKY17
Pbr018160.1	0.64	0.27	1.57^*^	1.11^*^	0.46	1.49^*^	WRKY17
Pbr020000.1	0.17	1.63^*^	−1.46^*^	1.99	4.47^*^	5.75^*^	WRKY18
Pbr011544.2	0.54	−0.4	−1.63	0.71	1.04^*^	1.18^*^	WRKY33
Pbr004885.1	0.39	−1.08	−0.18	0.35	0.43	0.4	WRKY40
Pbr019026.1	1.8^*^	3.01^*^	0.94	1.41	3.32^*^	3.61^*^	WRKY40
Pbr019030.1	−1.22^*^	−1.87^*^	0.8	−0.21	−0.59	1.73^*^	WRKY40
Pbr020001.1	−2.03^*^	−1.86^*^	−0.7	0.43	0.27	3.56^*^	WRKY40
Pbr022408.1	−0.49	−2.43	−1.36	1.39^*^	1.33^*^	2.19^*^	WRKY40
Pbr019883.1	−0.66	−0.7	0.26	−1.67^*^	−1.26	0.54	WRKY50
Pbr026903.1	0.67	1.24	−0.42	1.23	2.04^*^	4.69^*^	WRKY51
Pbr031922.1	0.73	−0.98	−0.31	0.9	1.52^*^	2.41^*^	WRKY6
Pbr022698.1	1.69^*^	1.75^*^	2.74^*^	1.06^*^	0.03	0.85	WRKY65
Pbr001424.1	0.77	0.6	0.59	0.93	1.32^*^	2.30^*^	WRKY70
Pbr002398.1	1.34	0.78	0.81	0.8	3.21^*^	4.48^*^	WRKY28
Pbr015149.1	1.48	−0.17	2.80^*^	2.53	4.09^*^	7.34^*^	WRKY75
Pbr042883.1	1.45^*^	0.34	0.65	0.98	2.40^*^	3.97^*^	WRKY75
**TRANSCRIPTION FACTORS–bZIP**							
Pbr002622.1	0.28	0.13	2.29	0.07	1.36^*^	1.27	bZIP
Pbr004364.1	−1.12	−0.35	−2.65	0.37	1.04^*^	1.08	BZO2H3
Pbr009262.1	0.26	−0.44	0.72	3.01	4.18^*^	7.23^*^	bZIP9
Pbr018534.1	0.90	0.60	−0.17	0.98	2.22^*^	2.20^*^	bZIP11-like
Pbr040479.1	0.85	0.08	−0.21	1.24^*^	1.56^*^	0.23	bZIP11-like

* *The gene was differentially expressed in the correspondent pairwise analysis (p ≤ 0.05)*.

### Genes involved in disease resistant proteins and defense response

We found that nine R genes encoding nucleotide-binding site-leucine-rich repeat (NBS-LRR) proteins were differentially expressed. Of these, ADR1-L1 (Pbr036409.1), and CC-NBS-LRR (Pbr016325.1) were differentially up-regulated at the early stage of infection in the susceptible genotype SC1 (Supplementary Table S3).

Based on K-mean results, defense responses were significantly up-regulated in SC1 (Figure 3C). DEGs related to defense responses were further analyzed. Genes encoding PR proteins exhibited similar expression patterns. For example, most genes were differentially up-regulated at the early stage of infection in the resistant genotype CG but the later stage of infection in the susceptible genotype SC1, i.e., PR1 (Pbr022550.1), PR5 (Pbr036399.1), EP3 (Pbr009767.1), and Chitinase A (Pbr018708.1), endo-1,3-beta-glucosidase 14 (Pbr001155.2) (Table 5).

**Table 5:** **Unigenes associated with defense response exhibiting a Log2 FC ≥ 1 and ***p*** ≤ 0.05 in at least one transition**.

**Gene ID**	**CG (Resistant)**			**SC1 (Susceptible)**			**Description**
	**FCC1/C0**	**FCC2/C0**	**FCC3/C0**	**FCS1/S0**	**FCS2/S0**	**FCS3/S0**	
**DEFENSE RESPONSE/PATHOGENESIS-RELATED PROTEINS**							
Pbr022550.1	1.63^*^	0.29	−0.19	−0.25	1.52^*^	1.77^*^	PR1
Pbr036399.1	3.22^*^	0.96	1.85^*^	0.01	5.05^*^	7.61^*^	PR5
Pbr009767.1	1.55^*^	0.08	−2.97	−1.05	1.07^*^	−0.06	EP3
Pbr009783.1	1.16	−0.41	1.88	−1.13	1.24^*^	3.16^*^	EP3
Pbr009781.1	1.50	0.20	4.23^*^	−0.98	1.37^*^	5.22^*^	EP3
Pbr027703.1	1.18	0.31	−3.56	1.64	3.16^*^	3.01^*^	PR3
Pbr007327.1	4.67	1.40	–	4.21	7.46^*^	7.72^*^	Chitinase II
Pbr001155.2	1.00^*^	−1.61	−2.02	−0.65	2.99^*^	4.19^*^	ß-1,3-BGL14
Pbr007327.1	4.67	1.40	–	4.21	7.46	7.72	CHI II
Pbr041409.1	1.05^*^	−0.02	1.90^*^	0.43	0.35	2.78^*^	BSP
Pbr039396.1	0.70	1.81^*^	2.67^*^	−1.39^*^	−0.13	1.50^*^	PRX2
**SECONDARY METABOLITES—SHIKIMATE ACID PATHWAY**							
Pbr006578.1	1.15^*^	−0.79	−2.65	0.24	2.56^*^	2.43^*^	Shikimate 5-dehydrogenase
Pbr042387.1	−0.59	−0.98	2.09^*^	−0.33	0.01	2.55^*^	Chorismate synthase
Pbr040661.1	−1.19	−1.06	2.27^*^	−0.64	−0.36	3.01^*^	EPSP synthase 2
Pbr029378.1	0.00	−0.12	3.55^*^	−0.09	0.18	3.16^*^	Prephenate dehydrogenase
Pbr003095.1	0.71	0.45	1.33	1.36	1.70	3.84^*^	Arogenate dehydrogenase
**SECONDARY METABOLISM-PHENYLPROPANOIDS**							
Pbr041924.1	4.20^*^	1.65	0.65	1.56	5.10^*^	6.45^*^	LAC7
Pbr012356.2	2.13^*^	−0.82	−1.19	2.42^*^	1.92^*^	0.65	BAHD acyltransferase
Pbr035966.1	1.08^*^	0.44	−0.90	0.91	2.55^*^	−0.14	BAHD acyltransferase
Pbr037017.1	1.39^*^	0.73	0.73	0.35	1.06^*^	2.12^*^	Kynurenine–oxoglutarae transaminase 1
Pbr040249.1	0.72	0.05	4.36^*^	1.61^*^	1.00	5.05^*^	Cinnamyl alcohol dehydrogenase 1
Pbr040236.1	0.57	0.01	−0.54	1.51^*^	0.77	0.62	Cinnamyl alcohol dehydrogenase 1
Pbr040244.1	0.75	0.25	1.62^*^	1.28^*^	0.77	2.82^*^	Cinnamyl alcohol dehydrogenase 1
Pbr011592.1	0.93	1.79^*^	1.35^*^	1.01^*^	0.12	0.21	Cinnamoyl-CoA reductase 1
Pbr008363.1	−0.17	−2.04	−4.19	0.73	2.24^*^	1.76^*^	PAL1
Pbr030350.1	0.75	−0.16	0.96	0.12	1.37^*^	2.66^*^	4-coumarate–CoA ligase-like 7
Pbr039972.1	−0.34	−1.83	−1.90	0.52	1.23^*^	2.37^*^	4-coumarate–CoA ligase-like 2
Pbr020454.1	1.40^*^	0.72	−1.09	−0.02	0.98	−0.55	SRG1
Pbr020456.1	1.38^*^	0.64	3.06^*^	0.40	1.18^*^	3.94^*^	SRG1
Pbr020457.1	1.16^*^	0.57	1.87^*^	0.38	1.15^*^	3.49^*^	SRG1
Pbr002584.2	1.09^*^	−0.74	−2.92	2.35	2.54^*^	1.03	SRG1
Pbr038607.1	2.44	3.99^*^	3.65^*^	2.67	5.66^*^	5.07^*^	Feruloyl CoA ortho-hydroxylase 2
Pbr004156.1	1.05^*^	−0.02	0.54	0.24	1.30^*^	1.72^*^	CYP736A12
Pbr007791.1	3.45^*^	0.52	0.45	0.85	1.54^*^	1.13	Caffeic acid 3-O-methyltransferase
Pbr037476.1	1.08^*^	−0.27	2.17	0.11	0.58	2.63^*^	Cationic amino acid transporter 1
**SECONDARY METABOLISM-FLAVONOIDS**							
Pbr021494.1	5.73^*^	2.70	−0.94	6.56	9.26^*^	5.24	CHS
Pbr021495.1	5.24^*^	2.65	−0.68	3.14	5.46^*^	1.31	CHS
Pbr027910.1	4.53^*^	2.27	4.03^*^	4.92	7.74^*^	8.85^*^	CHS
Pbr027907.1	3.54^*^	1.78	3.32^*^	3.32^*^	7.11^*^	6.20^*^	CHS
Pbr027916.1	4.38^*^	1.72	5.87^*^	2.23	4.86^*^	6.91^*^	CHS
Pbr029502.1	2.86^*^	−0.37	−1.92	2.82	6.08^*^	2.43	Flavonol synthase 3
Pbr007997.1	0.16	−1.64	−5.97	1.31^*^	1.96^*^	1.65^*^	Leucoanthocyanidin dioxygenase

* *The gene was differentially expressed in the correspondent pairwise analysis (p ≤ 0.05)*.

We further analyzed DEGs involved in secondary metabolism. All DEGs related to the secondary metabolism were classified into the Shikimate, phenylpropanoid, and flavonoid pathways. Most genes related to the Shikimate acid pathway were only differentially induced at 3 dpi either in CG or SC1, such as chorismate synthase (Pbr042387.1), EPSP synthase 2 (Pbr040661.1) and prephenate dehydrogenase (Pbr029378.1) (Table 5). Nineteen DEGs were related to the phenylpropanoid pathway. Seven DEGs were related to the flavonoid pathway. Of these, genes encoding chalcone synthase (CHS) (Pbr027907.1) and leucoanthocyanidin dioxygenase (Pbr007997.1) were differentially induced in the susceptible genotype SC1 leaves (Table 5).

### ET level and gene expression of hormone biosynthesis pathway

**Figure 5:**
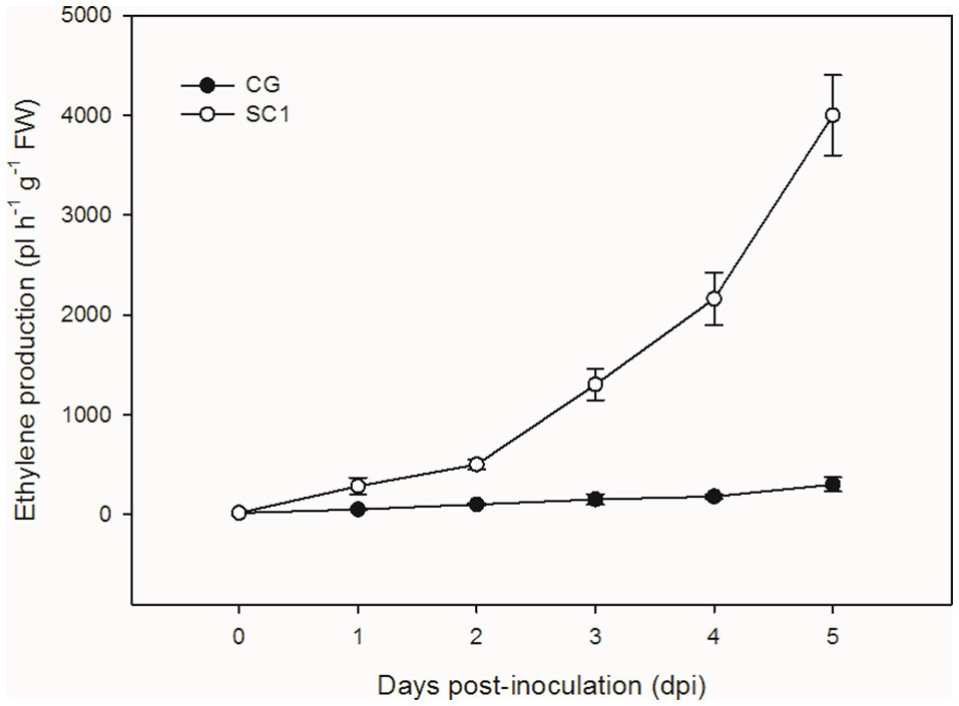
**Ethylene production**. Levels of ET were measured in inoculated leaves of CG and SC1 at given time points. Mean values are shown from five independent biological replicates [error bars, ±standard error (SE)] containing at least 20 leaves for every experiment.

**Figure 6:**
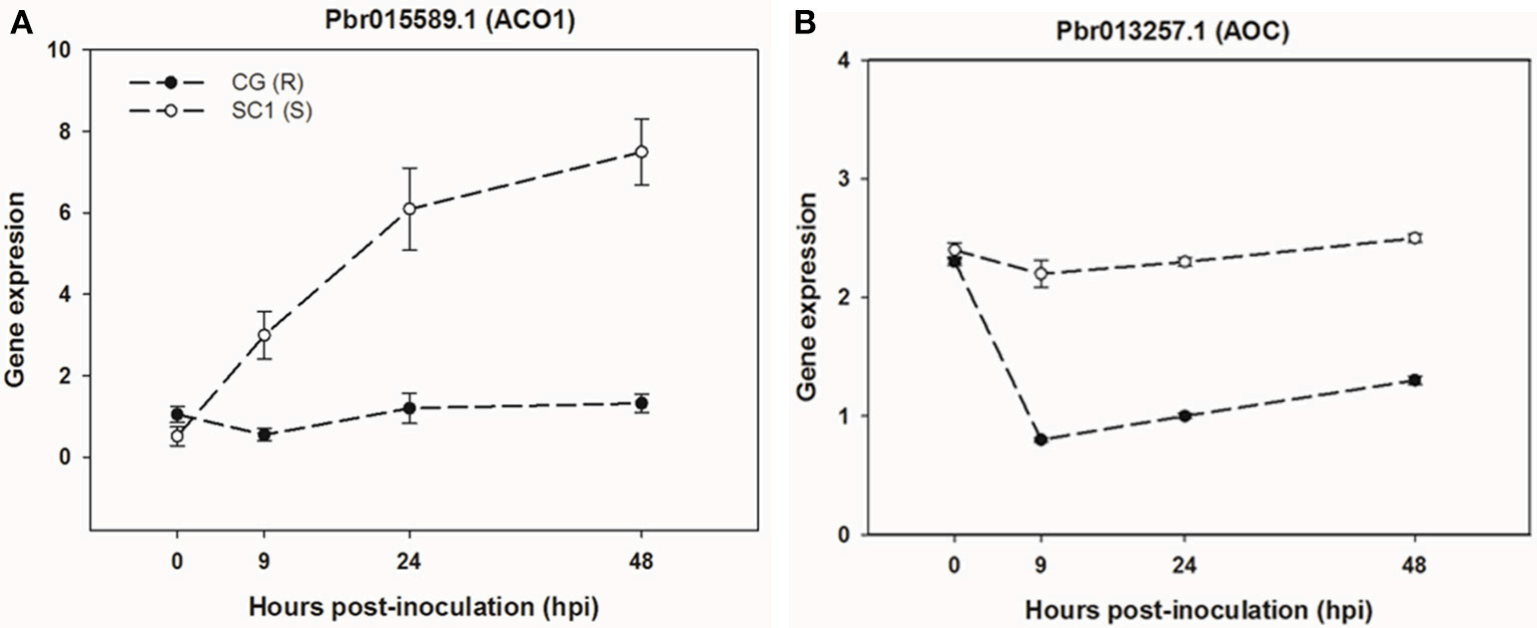
**Gene expression of ET biosynthesis gene ***ACO1*** (A)** and JA biosynthesis gene *AOC* **(B)** in sand pear cultivars CG and SC1 in response to *A. alternata* infections. Relative expression was obtained using qRT-PCR. *GAPDH* was used as an internal control. Mean values are shown from three independent biological replicates [error bars, ±standard error (SE)].

To further confirm the link between ET level and susceptibility response of pear to the pathogen, we determined ET levels in CG and SC1 leaves after inoculation (Figure 5). In infected leaves, ET levels were much higher in the susceptible genotype SC1 than in the resistant genotype CG. Levels of ET continuously increased after 1 dpi and peaked at 5 dpi in the SC1 leaves. In comparison, ET evolution was much slower in the resistant genotype CG. To further confirm the involvement of ET in the susceptibility response of pear to the pathogen, we monitored the expression of an *ACO1*-like gene at earlier time points post inoculation using qRT-PCR. The results showed that the expression of an *ACO1*-like gene was continuously higher in the susceptible genotype SC1 than in the resistant genotype CG (Figure 6A). Approximately 6-fold increases in the *ACO1*-like gene expression were detected in SC1 as early as 9 hpi, compared to 0 hpi. Approximately 11.94- and 14.69-fold increases were observed at 24 and 48 hpi in the susceptible genotype SC1. On the other hand, 0.52-fold decreases were found at 9 hpi, with 1.1-fold increases at 24 and 48 hpi in the resistant genotype CG (Figure 6A). In addition, to determine whether JA biosynthesis was involved in early response of pear to the pathogen, we performed gene expression analysis for a JA biosynthesis gene *AOC*. Expression of the *AOC* gene displayed 0.43-fold increases only at 2 dpi in the susceptible genotype SC1, while the gene expression was repressed in the resistant genotype CG (Figure 6B).

### H_2_O_2_ and antioxidant enzyme analysis

**Figure 7:**
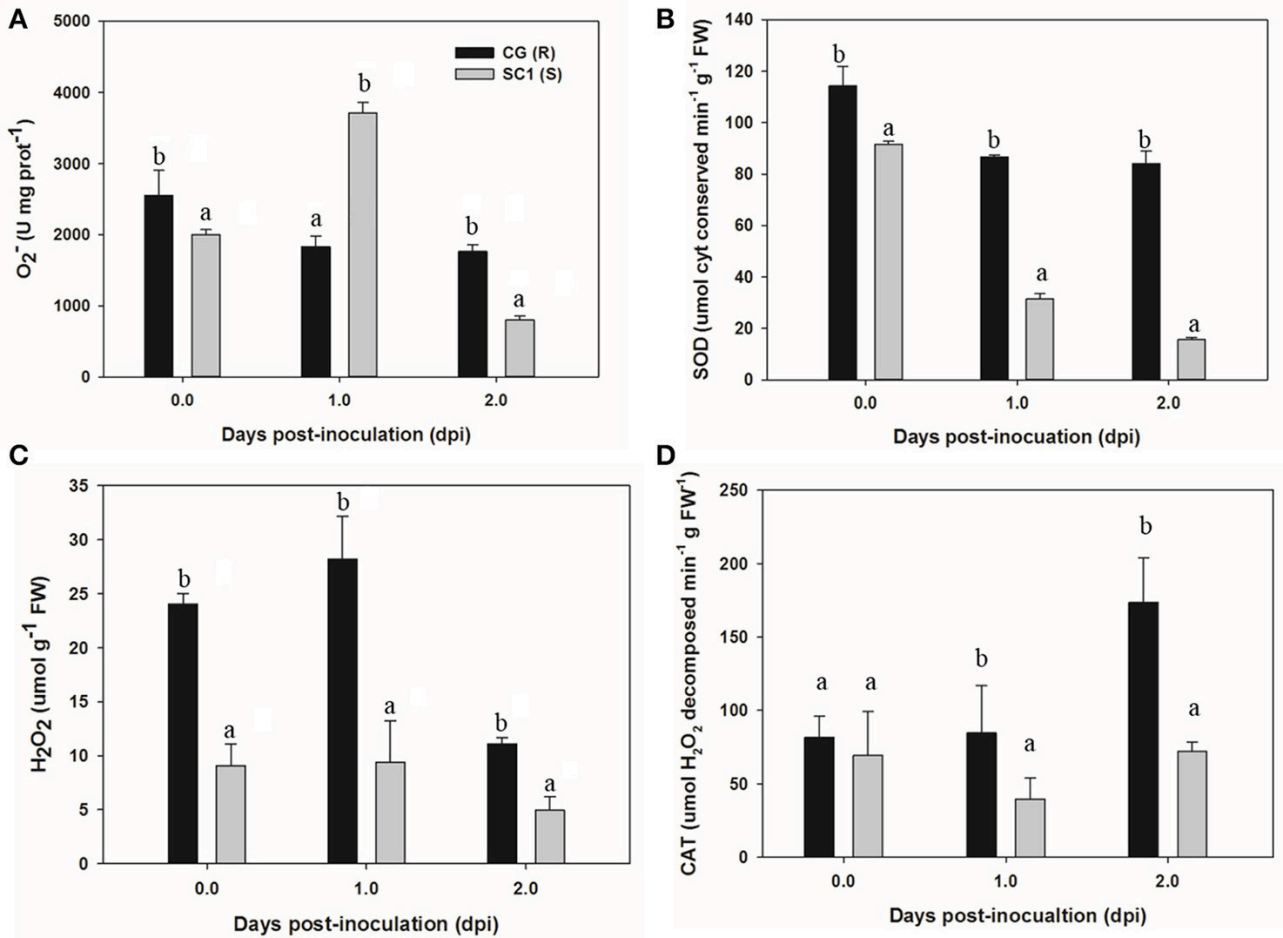
**Activities of H_**2**_O_**2**_ and antioxidant enzymes in sand pear cultivars CG and SC1 in response to ***A. alternata*** infections**. Levels of ${\text{O}}_{2}^{-}$ **(A)**, SOD **(B)**, H_2_O_2_ **(C)**, and CAT **(D)** were measured in inoculated leaves of CG and SC1 at given time points. Mean values are shown from five independent biological replicates [error bars, ±standard error (SE)] containing at least five leaves for every experiment. Different letters indicate significant differences between two genotypes at a given time point (*P* ≤ 0.05).

To acquire more information about whether H_2_O_2_ and antioxidant enzymes were involved in response of the susceptible and resistant genotypes pear to the pathogen, we determined the levels of ${\text{O}}_{2}^{-}$, SOD, H_2_O_2_, and CAT in the CG and SC1 leaves (Figure 7). The results showed that the activities of ${\text{O}}_{2}^{-}$ and SOD were induced in the susceptible genotype SC1 but repressed in the resistant genotype CG after inoculation (Figures 7A,B). The levels of H_2_O_2_ were increased by the pathogen infection in both genotypes of CG and SC1 (Figure 7C). CAT activity was increased significantly in the resistant genotype CG, but reduced significantly in the susceptible genotype SC1 (Figure 7D).

### Validation of transcriptome data using qRT-PCR

**Figure 8:**
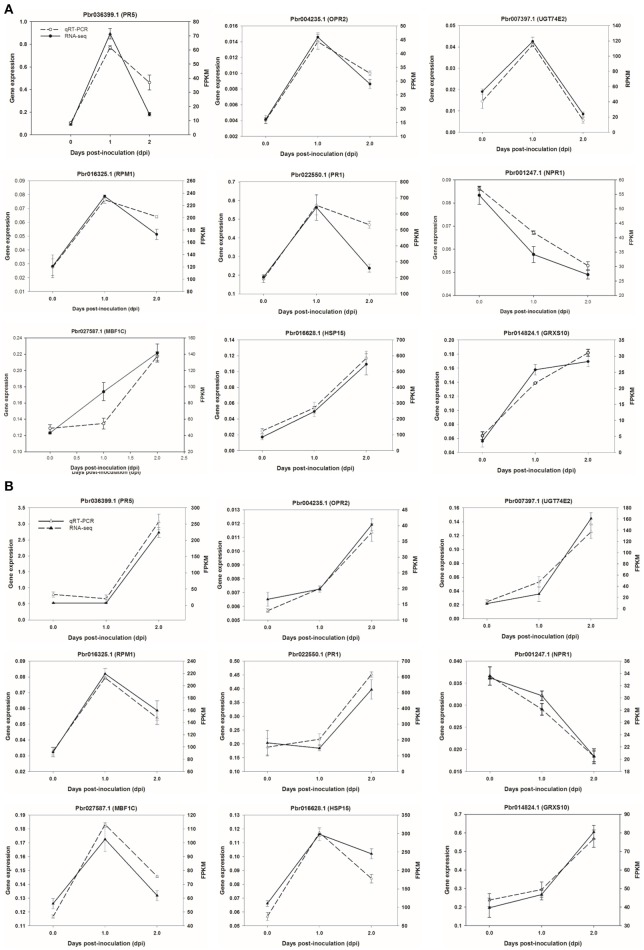
**Validation of RNA-seq data by quantitative real-time PCR**. Six genes were selected and their time course expression profiles were evaluated by quantitative real-time PCR in CG **(A)** and SC1 **(B)** at given time points. Relative expression was obtained using *GAPDH* as an internal control. cDNAs were synthesized from three biological replicates.

To validate RNA-seq data, we selected six genes to confirm the expression patterns by qRT-PCR using a house-keeping *GAPDH* gene as an internal control. As shown in Figure 8, results of qRT-PCR were consistent with the RNA-seq data either in CG or SC1, thereby validating the RNA-seq data.

## Discussion

### Hypersensitive response and programmed cell death during successful pathogen infections

Host cell death is a programmed event during interactions between plants and pathogens (Greenberg and Yao, 2004). Whether disease resistance or susceptibility is associated with PCD is dependent on the lifestyle of the pathogen (Greenberg and Yao, 2004). PCD plays a critical role in enhancing the growth of *A. alternata* f. sp. *lycopersici* (Greenberg and Yao, 2004). Herein, during the entire infection period, stronger symptoms of PCD in the susceptible cultivar SC1 than its male parental, resistant cultivar CG was illustrated by continuously and differentially up-regulated expression of PCD-related genes (Table 2), for example, PLP2. A positive relationship between *PLP2* expression and cell death was found in Arabidopsis (La Camera et al., 2009). *PLP2*-silenced plants are more resistant to *B. cinerea* and *P. syringae*. On the other hand, transgenic plants overexpressing *PLP2* exhibit enhanced susceptibility to these pathogens (La Camera et al., 2005). Significantly higher expression levels of the gene encoding PLP2 in SC1 than in CG suggested that PLP2 might facilitate necrotic symptoms in SC1, supporting by more lesion developments in the susceptible genotype SC1 than in the resistant genotype CG (Figure 1A).

### A predominant role of ET biosynthesis in PCD during successful pathogen infections

ET is produced by converting methionine to S-adenosylmethionine to 1-aminocyclopropane-1-carboxylic acid (ACC) via ACC synthase (ACS) and ACC oxidase (ACO), respectively (Kende, 1993). ET causes necrosis in plant tissues in response to unfavorable environmental and biotic stresses. Host-derived ET is considered to be an important signal for disease development (Bouchez et al., 2007). ET production is induced by several pathogen systems (Broekaert et al., 2006). The pathogen *A. alternata* cannot produce ET in culture but can induce higher accumulation of ET in infected plant tissues in cotton (Bashan, 1994). A positive correlation between the susceptibility of plants to *A. alternata* and ET levels suggests that ET could serve as a possible marker of susceptibility to *A. alternata* pv. *citri* (Ortuno et al., 2008). AAL toxin extracted from *A. alternata* f. sp. *lycopersici* induces ET production in tomato (Moussatos et al., 1994) and Arabidopsis (Gechev et al., 2004). Exogenous ET treatment results in enhanced disease development with necrotrophic pathogens (Abeles et al., 2012). In *B. cinerea*, application of ET synthesis inhibitors decreases susceptibility of plants to the pathogen (Abeles et al., 2012). In this study, we also observed the continuously up-regulated expression of ET biosynthesis genes ACS, ACO1, and ACO4, accompanied by a dramatic increase in ET production after inoculation in the susceptible genotype SC1 but not in the resistant genotype CG (Figures 5, 6 and Table 3).

**Figure 9:**
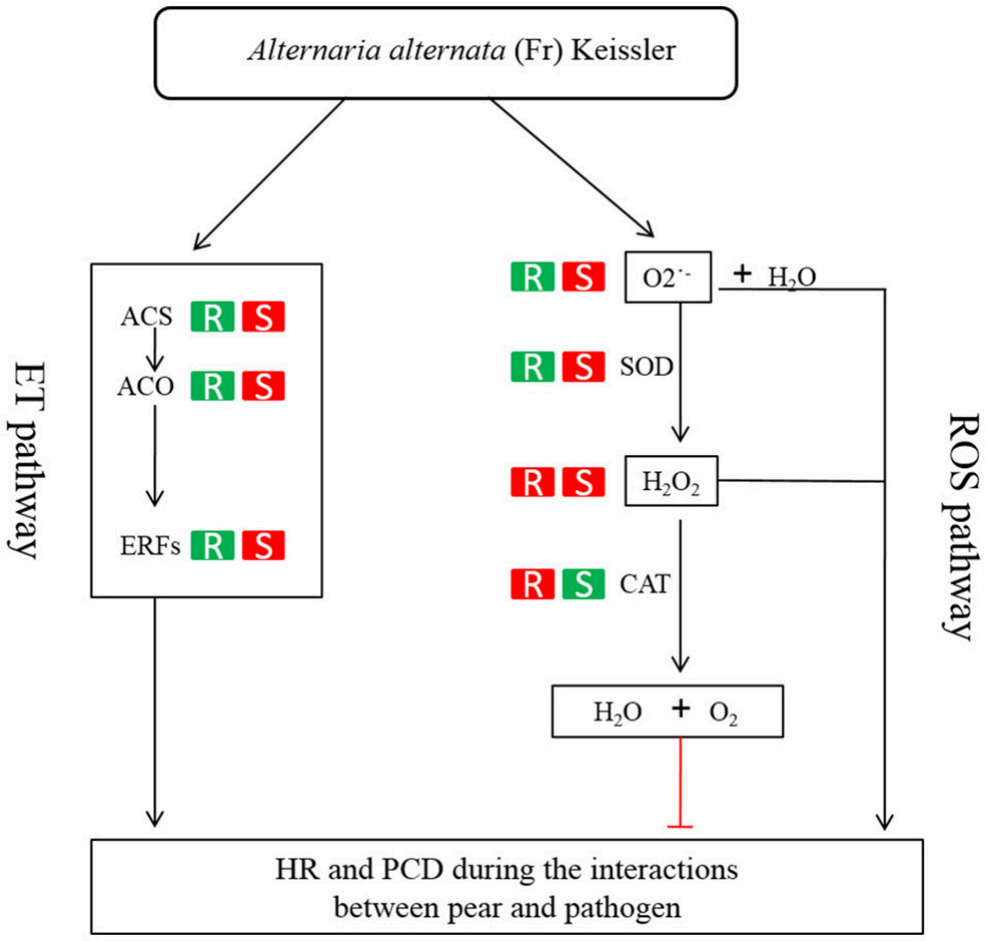
**Model of ET and H_**2**_O_**2**_ mediated pathways determining the compatible interaction of pear and ***A. alternata*****. When pear encounters the pathogen, ET biosynthesis (ACS, ACO) and ET signaling pathway (ERFs) are triggered and H_2_O_2_ and ${\text{O}}_{2}^{-}$ productions are induced. Consequently, HR and PCD occur and the successful infection is established in the susceptible genotype SC1. On the other hand, the ET biosynthesis and ET signaling pathway is repressed. The CAT activity is induced to decompose H_2_O_2_ into water and oxygen. Subsequently, the resistant response of pear to the pathogen is established in the resistant genotype CG. R, the resistant genotype CG; S, the susceptible genotype SC1; Red boxes, up-regulated expression or increased level of enzyme; green boxes, down-regulated expression or reduced level of enzyme.

ET is perceived by receptors such as ETR2 and ERS1 (Alexander and Grierson, 2002). ET signaling components such as *EIN2, EIN3, EIN4*, and *ERF1* are involved in the regulation of cell death and defense responses (Bouchez et al., 2007). In addition, previous studies demonstrated that MACD1, an APETALA2/ERF transcription factor, participates in AAL-triggered cell death (ACD) and acts in the downstream of ET signaling during ACD (Mase et al., 2013). The activation of ERF1 requires both ET and JA signaling pathways (Lorenzo et al., 2003). Similarly, ERF2 is induced by the pathogen *A. brassicicola*, MeJA, and ET (McGrath et al., 2005). Interestingly, ERF2 regulates the transcription of the genes related to the JA/ET-mediated defense response pathways such as Plant Defensin1.2 (*PDF1.2*) and pathogenesis-related protein 4 (*PR4*) (Ohme-Takagi and Shinshi, 1995; Brown et al., 2003). In this study, we found that the expression of ERF1 (Pbr001363.1) and ERF2 (Pbr035775.1) was differentially induced by the infection of the pathogen in the susceptible genotype SC1 leaves. However, genes encoding PDF1.2 (Pbr012199.1 and Pbr020807.1) and PR4 (Pbr009764.1, Pbr009765.1, Pbr009785.1, and Pbr009786.1) did not display sustained and differentially up-regulated expression in either CG or SC1 (Supplementary Table S3). Furthermore, our data revealed that other ERF genes such as PTI (Pbr016185.1), ERF113-like (Pbr029841.1), and RAP2-3 (Pbr012024.1) were highly activated by the pathogen in the susceptible genotype SC1 but not in the resistant genotype CG (Table 4). Taken together, our data suggest that ET biosynthesis and ET signaling pathway play a predominant positive role in PCD, attributing to the successful pathogen infection in the susceptible genotype SC1 (Figure 9).

### The role of antioxidant enzymes during pear-pathogen incompatible interactions

Studies suggest that ROS in the form of H_2_O_2_ and O_2_·^−^ are key mediators of PCD during HR (Bozhkov and Lam, 2011). The production of O2·^−^ is catalyzed by SOD (superoxide dismutase) into H_2_O_2_ (Bestwick et al., 1997). H_2_O_2_ is formed extra-cellularly and then diffuses into cells, resulting in the occurrence of PCD (Bestwick et al., 1997). CAT (catalase), as a key H_2_O_2_-detoxifying enzyme, decomposes H_2_O_2_ into molecular oxygen (O_2_) and water (H_2_O), maintaining leaf redox homeostasis (Wang Y. et al., 2013). The CAT mutants with reduced CAT activity display H_2_O_2_-induced leaf cell death phenotype in plants (Wang Y. et al., 2013). In this study, higher activities of O2^·−^, SOD and H_2_O_2_ suggested that ROS occurred at the early stage in the susceptible genotype SC1. H_2_O_2_ production was also triggered by the pathogen infection in the resistant genotype CG. However, increased CAT activity was detected in the resistant genotype CG but not in the susceptible genotype SC1. Our results demonstrate that the higher levels of CAT are likely to enhance the capability of cells to detoxify H_2_O_2_ and therefore repress H_2_O_2_-derived PCD during pear-pathogen interactions in the resistant genotype CG (Figure 9).

Notably, the expression of *CAT4* (Pbr001170.1) was not induced by the pathogen in either CG or SC1. Nevertheless, our results showed that genes encoding MBF1c (Pbr027587.1), HSP20 (Pbr016628.1), and Glutaredoxins10 (GRXS10) (Pbr014824.1) were significantly and continuously up-regulated in the resistant genotype CG but not in the susceptible genotype SC1 (Table 2). Previous researches demonstrate that MBF1c is a member of highly conserved transcriptional co-activator gene family which responds to oxidative stress (Arce et al., 2010). HSPs are induced by pathogen attack (Piterková et al., 2013). A positive relationship has been established between increased ROS production and HSPs expression. For example, *Oidium neolycopersici* infection stimulates HSP70 accumulation associated with increasing endogenous ROS levels in *S. chmielewskii* (Piterková et al., 2013). Glutaredoxins (GRX) are involved in the ROS-scavenging/antioxidant network. For example, the expression of GRXS13 restricts basal and high light stress-induced ROS production (Laporte et al., 2011). These results suggested that H_2_O_2_-derived PCD might be repressed by differentially up-regulated expression of MBF1c, HSP20, and GRXS10, and that higher H_2_O_2_ levels might be accumulated and redox homeostasis could be reached in the resistant genotype CG.

In addition, it is worth mentioning that several GO terms related to chloroplast and photosynthesis were significantly enriched. Analysis in detail showed that expression of those genes was increased after 2 dpi in the resistant genotype CG but decreased during the entire infection time in the susceptible genotype SC1 (Supplementary Table S3). The data suggested that the increases in photosynthetic processes may protect the photosynthetic apparatus against oxidative damage in the resistant genotype CG.

### Resistance responses of pear to *A. alternata* infection

The action of plant Resistance (R) genes belongs to the adaptive immune system in plant-pathogen interactions (Bonardi et al., 2011). When R genes recognize corresponding AVR genes, plant resistance to the pathogen is activated (Bonardi et al., 2011). In our transcriptome data, only two NBS-LRR genes (*RPM1* and *ADR1-L1*) were differentially up-regulated at 1 dpi in the susceptible genotype SC1. However, none were found in the infected resistant genotype CG leaf tissues (Supplementary Table S3). Yang et al. (2015) proposed that 28 candidate resistance genes with conserved leucine-rich repeats (LRR) domain might contribute to sand pear resistance to *A. alternata*. We found 23 of these genes in our transcriptome data. None of these genes were differentially expressed (Supplementary Table S3). The inconsistent results might be due to different cultivars or the biological samples collected from different time points. Our study aims to obtain a glimpse of early responses of sand pear to *A. alternata* attack. Interestingly, those candidate resistance genes referred by Yang et al. (2015) were not detected in the early response of sand pear to *A. alternata* in the study here. Further study is required to clarify the discrepancy.

The data presented here demonstrate that a compatible reaction in the susceptible genotype SC1 to *A. alternata* infection is established early during the interaction. Higher ET production and ROS levels culminate the occurrence of PCD and necrotic cells, promoting pathogen development in the susceptible genotype SC1. However, the differential expression of genes related to HR and the induced higher levels of CAT in the resistant genotype CG lead to reduced ROS accumulation and limited the growth of necrotic cells, therefore resulting in an incompatible process with resistance. Our study demonstrates that ET biosynthesis and ET signaling pathways and detoxifying of H_2_O_2_ determine whether the interaction of sand pear and *A. alternata* is incompatible or compatible (Figure 9).

## Author contributions

HW, JL and CJ conceived and designed the experiments. HW performed the experiments. HW and CJ analyzed the data. JL and YC contributed reagents/materials/analysis tools. HW and CJ wrote the manuscript.

## Funding

This work was supported by the National Natural Science Foundation of China (Grant No. 31540052), the Jiangsu Natural Science Foundation of China (Grant No. BK 20141383) and the Jiangsu Agricultural Science and Technology Independent Innovation Fund of China [Grant No. CX (14)5016]. The funders had no role in study design, data collection and analysis, decision to publish, or preparation of the manuscript.

### Conflict of interest statement

The authors declare that the research was conducted in the absence of any commercial or financial relationships that could be construed as a potential conflict of interest.
