# Supplementary material for: Comparative Transcriptomic Analysis Reveals That Ethylene/H2O2-Mediated Hypersensitive Response and Programmed Cell Death Determine the Compatible Interaction of Sand Pear and Alternaria alternata
Source: Front Plant Sci. 2017 Feb 15;8:195. doi: 10.3389/fpls.2017.00195 (PMC5309250; doi:10.3389/fpls.2017.00195)
Supplement: Supplementary file 1 [file DataSheet1.docx]

Supplementary Table S1. Gene-specific primers for quantitative real-time PCR.

| Primer Name | Sequence(5'to3') |
| --- | --- |
| Pbr016325.1-F | GCCACCCCACCAGTTGACT |
| Pbr016325.1-R | GATGCCCTTCTTTTGGACAAGT |
| Pbr013257.1-F | CTCTCACTCCTTCCCAACCCT |
| Pbr013257.1-R | GAGGTGGTGAAGTTGCTGGAG |
| Pbr004235.1-F | CCCAGTTTGTAGGGAGTAAGGA |
| Pbr004235.1-R | CCAAGATTAGGTCTGACAACGG |
| Pbr015589.1-F | GCTTGTGAGAGGACTTCGGG |
| Pbr015589.1-R | TTGGTCATCTTGGAGCAGGAG |
| Pbr007397.1-F | AAATGGAGCCCTCAGTTGGA |
| Pbr007397.1-R | CGCCGTGTGTGAAAAAACAC |
| Pbr036399.1-F | TGGGGCAACAGACATAGGC |
| Pbr036399.1-R | AGCAAACGGGGGTCAAGAT |
| Pbr022550.1-F | GCGGAGAAAGCCGACTACAA |
| Pbr022550.1-R | CACCCTACACGAGCCGAGTTAC |
| Pbr001247.1-F | TGATTGCCTACGTCAGATGG |
| Pbr001247.1-R | AATGGTGCTTGGACGAACTC |
| Pear-GAPDH-F | TGGTGTGAACGAGAAGGAAT |
| Pear-GAPDH-R | CCCTCAACAATCCCAAACC |

Supplementary Table S2. Number of differentially expressed genes between uninoculated and inoculated samples and two genotypes at given time points.

| Sample | C0 | C1 | C2 | C3 | C5 | S0 | S1 | S2 | S3 | S5 |
| --- | --- | --- | --- | --- | --- | --- | --- | --- | --- | --- |
| C0 | NA |  |  |  |  | 1188 |  |  |  | - |
| C1 | 951 |  | 761 |  |  |  | 1154 |  |  | - |
| C2 | 1134 |  |  | 795 |  |  |  | 1414 |  | - |
| C3 | 5834 |  |  |  | 493 |  |  |  | 3114 | - |
| C5 | 6000 |  |  |  |  |  |  |  |  | 2623 |
| S0 |  |  |  |  |  | NA |  |  |  |  |
| S1 |  |  |  |  |  | 1040 |  | 885 |  |  |
| S2 |  |  |  |  |  | 1319 |  |  | 826 |  |
| S3 |  |  |  |  |  | 7222 |  |  |  | 811 |
| S5 |  |  |  |  |  | 7526 |  |  |  |  |

Supplementary Table S3 Fold change of unigenes associated with related biological function

| Gene ID | CG (Resistance) | | | SC1 (Susceptibility) | | | Description |
| --- | --- | --- | --- | --- | --- | --- | --- |
|  | FCC1/C0 | FCC2/C0 | FCC3/C0 | FCS1/S0 | FCS2/S0 | FCS3/S0 |  |
|  |  |  |  |  |  |  |  |
| Pbr025376.1 | 0.17 | 0.51 | 0.57 | 0.45 | -0.08 | -2.50 | disease resistance protein |
| Pbr025080.1 | -0.25 | -0.86 | -2.02 | 0.71 | -0.26 | 0.96 | disease resistance protein |
| Pbr022876.1 | 0.14 | 0.46 | -2.02 | 0.40 | 0.52 | -2.69 | disease resistance protein |
| Pbr023278.1 | -0.70 | 0.02 | 3.06 | 0.19 | -0.22 | 0.24 | disease resistance protein |
| Pbr012791.1 | 0.13 | 0.05 | -3.79 | 0.03 | -0.42 | -2.78 | disease resistance protein |
| Pbr022889 | - | - | - | - | - | - | disease resistance protein |
| Pbr033741.3 | -0.07 | -0.07 | -1.25 | 0.45 | -0.03 | -0.59 | disease resistance protein |
| Pbr022874.1 | -0.02 | -0.26 | -2.92 | 0.69 | 0.81 | -2.46 | disease resistance protein |
| Pbr023136.1 | -0.71 | -0.03 | 0.18 | 0.00 | -0.67 | 0.13 | disease resistance protein |
| Pbr007974.1 | 0.57 | 0.53 | -0.12 | 0.49 | 0.53 | -1.12 | disease resistance protein |
| Pbr040608.1 | -3.28 | -1.19 | -2.38 | -1.91 | -1.73 | -1.87 | disease resistance protein |
| Pbr008283.1 | 0.17 | 0.37 | -0.99 | -0.05 | -0.48 | -3.51 | disease resistance protein |
| Pbr041724.1 | -0.02 | 0.12 | -1.43 | 0.28 | -0.09 | -0.76 | disease resistance protein |
| Pbr023112.3 | -0.30 | -0.05 | -0.44 | -0.45 | 0.76 | -1.20 | disease resistance protein |
| Pbr039001.1 | -1.81 | -0.04 | 0.73 | -1.26 | -1.82 | -4.67 | disease resistance protein |
| Pbr000678.1 | -0.06 | 0.37 | -0.07 | 0.05 | -0.30 | -0.05 | disease resistance protein |
| Pbr000681.1 | -1.78 | -0.92 | -1.67 | -0.44 | -1.40 | -4.51 | disease resistance protein |
| Pbr012606.1 | -0.35 | -0.10 | - | 1.11 | -0.02 | -0.75 | disease resistance protein |
| Pbr001627.1 | - | - | - | 0.67 | 0.35 | -0.12 | disease resistance protein |
| Pbr034022.1 | -0.41 | -0.63 | -0.72 | 0.19 | -0.12 | 0.22 | disease resistance protein |
| Pbr012560.2 | 0.75 | 0.44 | -0.10 | 0.41 | 0.31 | 0.25 | disease resistance protein |
| Pbr038352.1 | -0.04 | -0.44 | -0.65 | -0.62 | -0.89 | -1.46 | disease resistance protein |
| Pbr035730.1 | 0.06 | 0.27 | -0.74 | -0.04 | -0.78 | -2.54 | disease resistance protein |
| Pbr001247.1 | -0.67 | -0.97 | -0.91 | -0.24 | -0.67 | 0.10 | disease resistance protein |
| Pbr004621.1 | -1.75* | -1.40* | -1.24* | -1.18* | -0.52 | -0.97 | disease resistance protein |
| Pbr009332.1 | -1.32* | -1.41* | -1.46* | -1.18* | -5.78* | -3.99* | disease resistance protein |
| Pbr016325.1 | 0.95 | 0.52 | -0.13 | 1.80* | 0.94 | 0.98 | CC-NBS-LRR |
| Pbr016327.1 | -1.03* | 0.01 | -1.46* | 0.60 | 1.22 | 1.12* | disease resistance protein |
| Pbr025086.1 | -0.50 | -0.28 | -0.56 | -1.16* | -1.73* | -2.69* | disease resistance protein |
| Pbr025091.1 | -1.23* | -1.45* | -2.37* | -0.84 | 0.13 | -1.36* | disease resistance protein |
| Pbr025092.1 | -1.30* | -1.51* | -3.02* | -1.30* | 0.16 | -1.53* | disease resistance protein |
| Pbr026230.2 | -0.63 | -0.83 | -0.25 | -1.36* | -1.06* | -2.34* | disease resistance protein |
| Pbr036409.1 | 0.67 | 0.50 | -1.27 | 1.04* | 0.38 | 0.04 | ADR1-L1 |
| Pbr011477.1 | -0.55 | 0.55 | -1.05* | -0.02 | -0.66 | -1.25* | ICS1 |
| Pbr006804.1 | -0.56 | -0.90 | -1.16* | -0.48 | -0.15 | 0.29 | EDS2 |
| Pbr006805.1 | - | - | - | - | - | - | EDS2 |
| Pbr008020.1 | -0.34 | -0.15 | -1.89 | 0.30 | 0.21 | -0.78 | EDS2 |
| Pbr010179.1 | - | - | - | - | - | - | EDS2 |
| Pbr015647.1 | -0.56 | -0.62 | 0.22 | -0.65 | 0.00 | 0.71 | EDS2 |
| Pbr019960.1 | 0.10 | 0.21 | 0.44 | 0.12 | 0.03 | -1.84 | EDS2 |
| Pbr029171.1 | -0.08 | 0.23 | -2.00 | -0.27 | 0.14 | -2.71 | EDS2 |
| Pbr034291.1 | 0.14 | 0.36 | 2.06 | -0.21 | 0.18 | 1.97 | EDS2 |
| Pbr036467.1 | -1.75 | -0.01 | -2.72 | -1.81 | -0.80 | -1.84 | EDS2 |
| Pbr039068.1 | -1.27 | -0.78 | -2.85 | -0.10 | -1.77 | -6.05 | EDS2 |
| Pbr012199.1 | - | - | - | -0.51 | - | - | PDF1 |
| Pbr020807.1 | - | - | - | - | - | - | PDF1 |
| Pbr009764.1 | -2.16 | -2.31 | -3.57 | 0.84 | 1.23 | -0.37 | PR4 |
| Pbr009765.1 | -1.59 | -1.84 | 0.47 | 0.80 | 1.32 | 2.44 | PR4 |
| Pbr009785.1 | -1.88 | -2.13 | 0.63 | 1.00 | 1.27 | 2.80 | PR4 |
| Pbr009786.1 | -1.65 | -2.07 | 0.51 | 0.62 | 1.07 | 1.95 | PR4 |


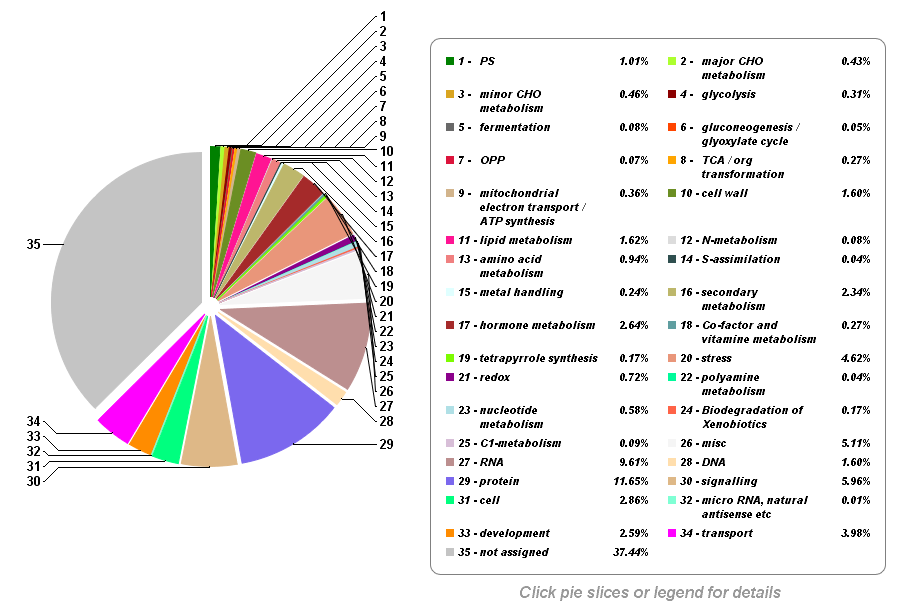


Fig. S1. Mapman functional annotations of the transcriptome. PS: photosynthesis, OPP: misc:

A
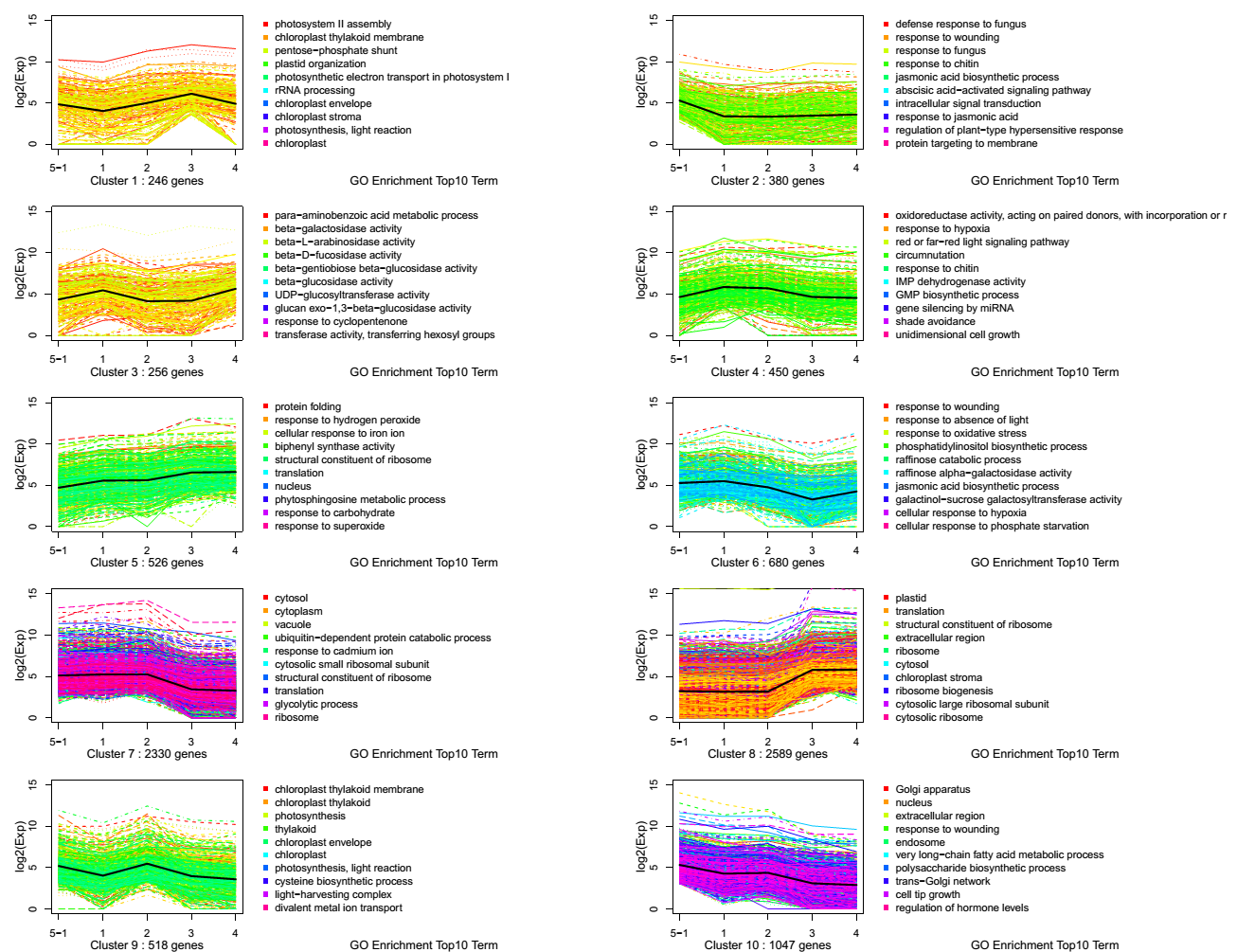


B
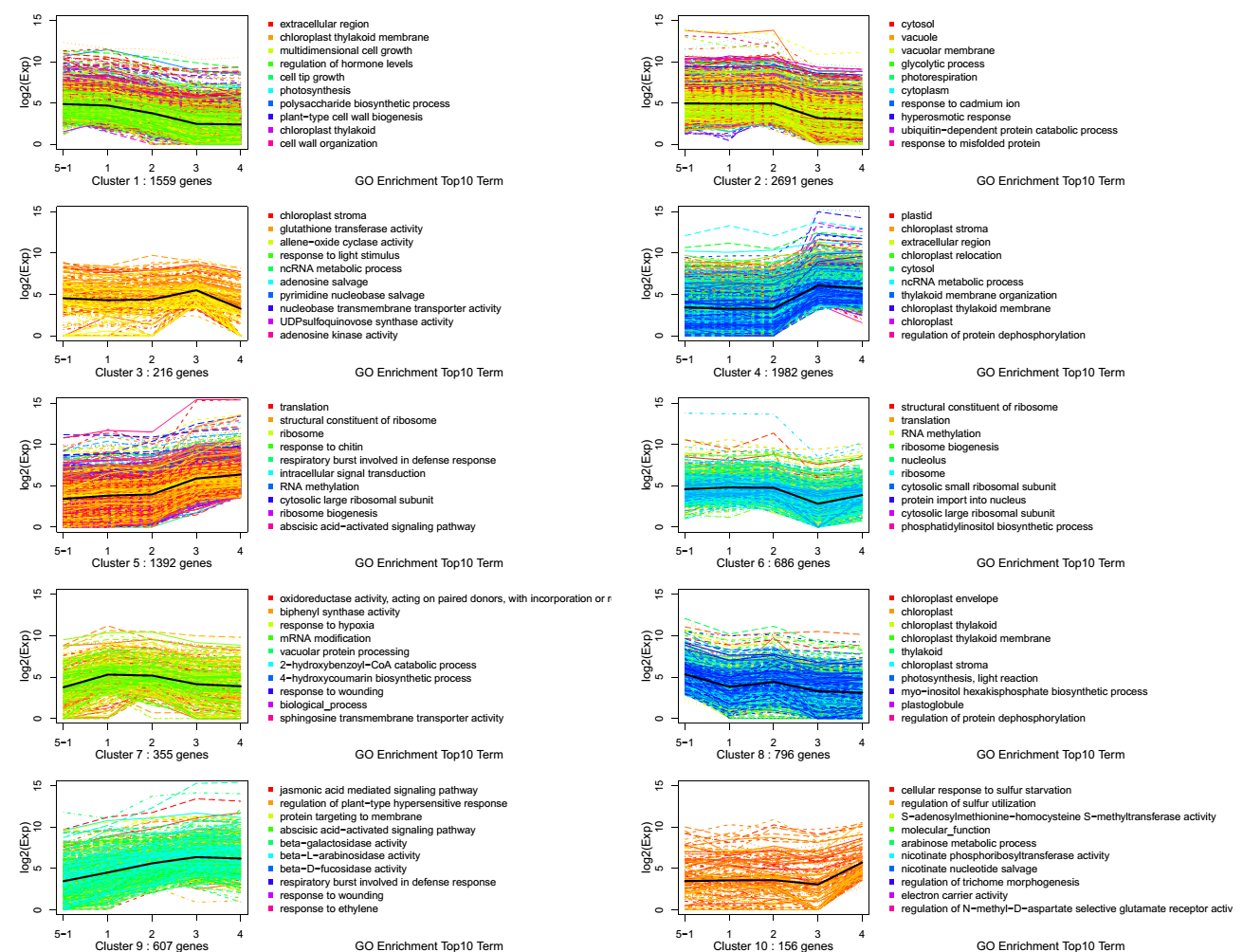


Figure. S2. Dynamic expression profiles of genes with top 10 GO term of two different genotypes by k-mean analysis. A. A, CG; B, SC1.


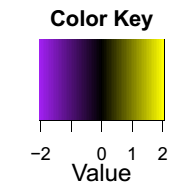

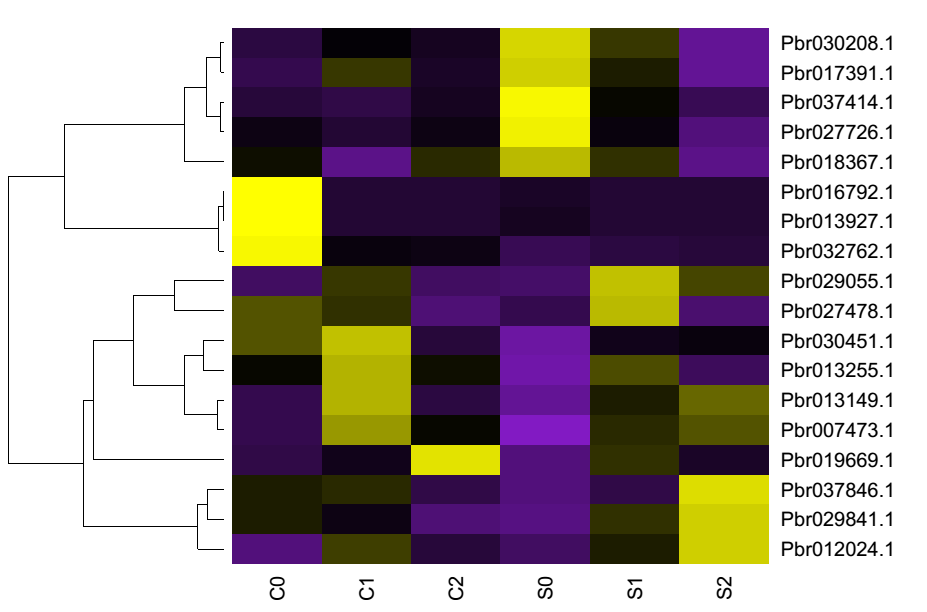


Figure.S3 Heat-map depicting the changes of ERFs genes expression in CG and SC1 with pathogen infection.

igure.S3 Heat-map depicting the changes of ERFs genes expression in CG and SC1 with pathogen infection.


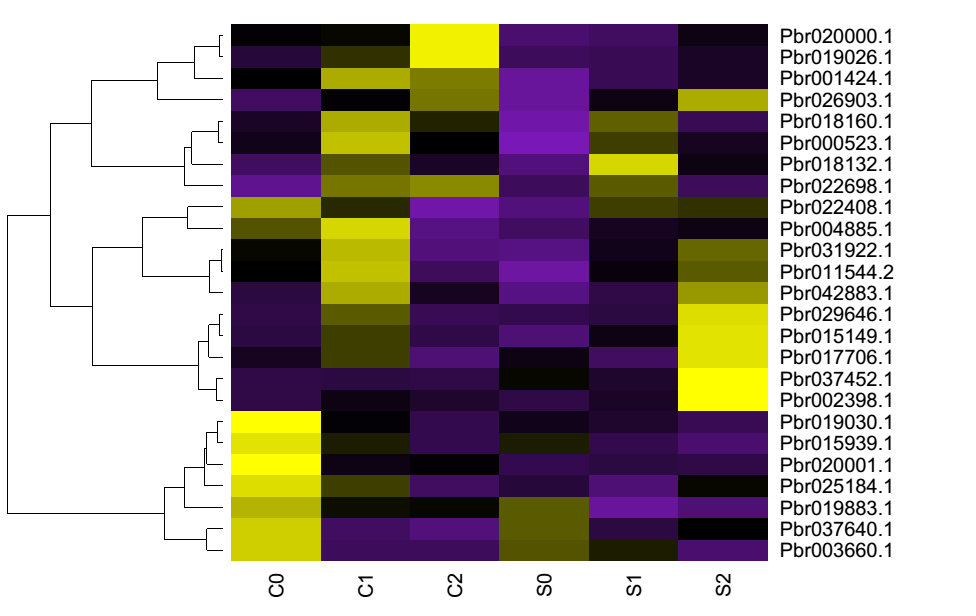

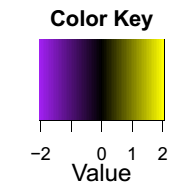


Figure.S4 Heat-map depicting the changes of WRKY genes expression in CG and SC1 with pathogen infection.
